# *Polyalthia longifolia* Extract Triggers ER Stress in Prostate Cancer Cells Concomitant with Induction of Apoptosis: Insights from *In Vitro* and *In Vivo* Studies

**DOI:** 10.1155/2019/6726312

**Published:** 2019-11-13

**Authors:** Saheed O. Afolabi, Olufunke E. Olorundare, Abiola Babatunde, Ralph M. Albrecht, Mamoru Koketsu, Deeba N. Syed, Hasan Mukhtar

**Affiliations:** ^1^Department of Pharmacology and Therapeutics, University of Ilorin, Ilorin, Nigeria; ^2^Department of Hematology, University of Ilorin, Ilorin, Nigeria; ^3^Department of Animal Sciences, 1046 Animal Sciences Building, University of Wisconsin, Madison, USA; ^4^Department of Chemistry and Biomolecular Science, Faculty of Engineering, Gifu University, Gifu, Japan; ^5^Department of Dermatology, University of Wisconsin, Madison, USA

## Abstract

Plant-based therapies are being explored to prevent or treat several cancer types. The antioxidant properties of *Polyalthia longifolia* plant are well established. In our previous work, we demonstrated the presence of cytotoxic compounds in the methanol extract of *Polyalthia longifolia* (MEP) with potent activity against human leukemia cells. In the present study, we evaluated the efficacy of MEP against prostate cancer (PCa) and established the molecular basis of its effect in *in vitro* and *in vivo* models. We observed that MEP treatment resulted in a significant decrease in the growth and viability of PCa cells, associated with arrest in the G1/S phase of the cell cycle. Apoptosis was confirmed as the primary mode of MEP-induced cell death through activation of the intrinsic apoptotic machinery. Proteomic and biochemical studies identified BiP as an important target of MEP with the activation of the ER stress pathway, as a potential mechanism driving MEP-induced apoptosis. The extract exhibited strong efficacy in the PCa xenograft mouse model with significant inhibition of tumor growth and reduced tumor burden. Taken together, our findings indicate that MEP-induced apoptosis in PCa cells concomitant with the activation of the ER stress pathways results in the inhibition of tumor growth, *in vitro* and *in vivo*. Our studies provide initial evidence of the efficacy of MEP against PCa and advocate for in-depth studies in other preclinical models for its possible use in clinical settings.

## 1. Introduction

Even with the advent of better treatment options, prostate cancer (PCa) remains the second most common cancer globally and a leading cause of cancer-related death in men [1, 2]. A total of about 1,735,350 new cancer cases and 609,640 cancer-related deaths were projected to occur in the United States in 2018 [3]. For the US alone, it is predicted that 164,690 new cases of PCa will be diagnosed in the year 2018 and an estimated 29,430 people will die of the disease. (https://seer.cancer.gov/statfacts/htmL/prost.html). Screening for PSA combined with digital rectal examination, needle biopsy, etc. has undoubtedly improved patient's survival through detection of early localized disease; the cure for advanced and metastatic PCa disease however still remains elusive [4].

One of the early changes in prostate tumorigenesis is a major remodeling of the cancer cell proteome associated with increases in protein biosynthesis [5]. Increased protein turnover and the ensuing flux in the endoplasmic reticulum (ER) creates a state of proteotoxic stress, accumulation of misfolded proteins, and activation of the unfolded protein response (UPR). The three signaling arms of UPR are composed of (i) ATF6 (activating transcription factor 6) which promotes ER homeostasis, (ii) IRE1 (inositol-requiring enzyme 1) which controls splicing of the transcription factor XBP1, and (iii) PERK (PKR (RNA-activated protein kinase)-like ER-associated protein kinase), which promotes downstream phosphorylation of eIF2*α* and directly regulates protein synthesis. Even though the precise combinations of oncogenes that control the distinct arms of the UPR pathway are still being studied, the association between ER stress, UPR activation, and neoplastic progression is well recognized [6–9]. Deletion of the tumor suppressor PTEN and increased activation of the oncogene MYC are present in nearly 50% of metastatic PCa [10]. Prostate tumors with combined PTEN loss and enforced MYC expression have reduced protein synthesis compared to tumors harboring either alteration alone. It was surmised that the reduced protein amounts might be a cytoprotective response to limit ER stress and facilitate tumor progression [11].

Targeting ER homeostasis is emerging as a new therapeutic strategy in PCa [9, 12]. Several small molecule drugs and chemical extracts that disrupt ER homeostasis in PCa cells are being explored [12, 13]. The anticancer activity of selenium and its metabolites on PCa cells is mediated at least in part, through activation of ER stress and subsequent induction of apoptosis [14]. The antidiabetic drug, metformin, decreases PCa risk in people by activating the miR-708-5p/neuronatin pathway, which subsequently leads to ER stress-induced apoptosis [9, 15]. In addition to enzyme inhibitors, dietary compounds were shown to trigger ER stress and induce apoptosis in PCa [9]. In this context, there is considerable evidence that diet, physical activity, and body weight management are critical to cancer progression and may serve as a yardstick for cancer recurrence [16]. Dietary schemes comprising of legumes, vegetables, fruits, unprocessed cereals, nuts, olive oil, etc. have been associated with reduced mortality after a prior diagnosis of nonmetastatic PCa [17]. *Polyalthia longifolia* also referred to as mast tree belongs to the *Annonaceae* family comprising over 120 species of shrubs and trees. *Polyalthia longifolia* is found in the tropic and subtropic regions [18]. Various parts of the plant have been used for the treatment of fever, skin diseases, diabetes, hypertension, and helminthiasis [19, 20]. Leaf extracts of *Polyalthia longifolia* reportedly possess antioxidant and radical scavenging properties [21]. It was shown that livers of extract-treated mice were protected against paracetamol-induced oxidative damage [21, 22]. Anti-inflammatory, antimicrobial, and antitumor activities of *Polyalthia longifolia* have also been reported [23–26]. Moreover, compounds including cycloartane, triterpenes, clerodane diterpene, tetranorditerpene, and methyl-tetranorditerpene isolated from plant leaves displayed marked growth inhibitory activity in *in vitro* studies against cancer cell lines [27, 28]. We showed previously that the leaf extract was effective against human leukemia cell lines [29]. Recent findings showed that Polyalthia longifolia induced apoptosis in cervical cancer HeLa cells via the regulation of miRNA, works synergistically with ampicillin against Methicillin-Resistant Staphylococcus Aureus (MRSA) and possesses antiplasmodial activity against chloroquine-sensitive malaria parasite strain NF54 with minimal toxicity to human red blood cells [30–32]. In this study, we further explored the antiproliferative potential of the methanol leaf extract of *Polyalthia longifolia* (MEP) with the aim of delineating its effect on PCa cell proteome and deciphering its mechanistic targets, employing both *in vitro* and *in vivo* study models.

## 2. Materials and Methods

### 2.1. Plant Material

Leaves of *Polyalthia longifolia* were collected from a residential apartment in Ilorin, Kwara State, Nigeria, between August 2015 and September 2016. The plant was identified and authenticated by Prof. Felix Oladele, a plant botanist from the Department of Botany, University of Ilorin, Ilorin, Nigeria, and a voucher specimen number: UILH/005/872 was deposited in the University Herbarium.

### 2.2. Reagents and Antibodies

All primary antibodies were purchased from Cell Signaling Technology. Anti-mouse and anti-rabbit secondary antibody horseradish peroxidase conjugates were obtained from Amersham Pharmacia Life Sciences. The Bio-Rad DC Protein Assay Kit was purchased from Bio-Rad; CA Novex precast Tris-Glycine gels were obtained from Invitrogen. The Annexin-V-FLUOS Staining Kit was purchased from Roche.

### 2.3. Cell Lines

PC3, DU145, C4-2, and PC3M-LUC-C6 human prostrate carcinoma cells were purchased from ATCC (American Type Culture Collection) and grown in RPMI 1640 (Life Technologies, NY) supplemented with 10% FBS and 1% penicillin/streptomycin, with 5% CO_2_, at 37°C. MEP dissolved in DMSO was used for the treatment of cells. Cells at a confluency of ~70% were treated with MEP at 10–100 *μ*g/mL for 24 h in complete cell medium where the final concentration of DMSO used for each treatment was less than 0.1% (*v*/*v*).

### 2.4. Plant Extraction

Air-dried leaves (710 g) were powdered and macerated in methanol at room temperature for 7 days. Successive maceration was carried out to exhaust the methanol constituent of the leaves. The filtrate from the successive maceration was concentrated in vacuo to give a final weight of 99.9 g and 14.1% yield.

### 2.5. Cell Viability Assay

3-(4,5-Dimethylthiazol-2-yl)-2,5-diphenyl tetrazolium bromide (MTT) assay was employed to study the effect of MEP on the viability of PC3, DU145, C4-2, and PC3M-LUC-C6 PCa cell lines. Cells were plated (1 × 10^4^ cells per well) in 1 mL of complete culture medium containing 10–200 *μ*g/mL concentrations of MEP in 24-well microtiter plates. After incubation in a humidified incubator for 24 h at 37°C, 200 *μ*L of MTT (5 mg/mL: 1x PBS) was added to each well and incubated for two hours, after which 200 *μ*L of DMSO was added. The plates were then centrifuged (1800 ×g for 5 min at 4°C), and absorbance at 540 nm was recorded on a microplate reader. The effect of MEP on growth inhibition was calculated as % cell viability.

### 2.6. Colony Formation Assay

Both DU145 and PC3M-LUC-C6 cells were treated with MEP (10 and 20 *μ*g/mL) in RPMI-1640 complete medium. Following treatment, 5000 cells/well were replated in triplicate on a 6-well tissue culture plate with and cultured in 5% CO_2_ at 37°C for 8 days with growth media being replaced with/without MEP every 2 days. The cells were then stained with 0.5% crystal violet (in methanol : H_2_O; 1 : 1), and pictures were taken using a digital camera. The colony intensity was measured using the ImageJ software.

### 2.7. Cell Cycle Analysis/Apoptosis by Flow Cytometry

Methods described by Shabbir et al. [33] were employed. DU145 and PC3M-LUC-C6 cells treated with MEP (20 and 40 *μ*g/mL: 24 h) in complete medium were trypsinized and fixed in 1% paraformaldehyde: 1x PBS for an hour, washed twice with cold PBS, and suspended in chilled 70% ethanol. Next day, cells were centrifuged for 5 min at 1000 rpm and the pellet obtained was washed twice with cold PBS to remove ethanol. The cells were labeled with FITC and propidium iodide using the Apo-Direct Kit (BD Pharmagen, CA) as per manufacturer's protocol. Analysis was performed with a FACScan (Becton Dickinson, NJ). About 10,000 events per sample were collected, and the DNA histograms were analyzed with ModFitLT software (Verily Software House, ME).

### 2.8. Protein Extraction and Western Blot Analysis

After treatment with MEP, ice-cold lysis buffer (Cell Signaling Technology, Danvers, MA) was added to the cells along with protease inhibitors (Calbiochem, Germany). Cells were homogenized by passing through a 23-gauge needle and centrifuged, and the supernatant was quantified for protein concentration. 40 *μ*g-60 *μ*g of protein were resolved on 8–12% polyacrylamide gels, transferred on to a nitrocellulose membrane, probed with appropriate primary and secondary antibodies, and detected by chemiluminescence autoradiography.

### 2.9. Sample Preparation and LC/MS/MS Analysis

For quantitative proteomics analysis, methods designed by Singh et al. [34] were employed. PC3 cells were treated with vehicle or MEP (20 *μ*g/mL) for 24 h in triplicate. The experiment was repeated to yield a total of 6 replicates. Treated cells were collected by trypsin digestion followed by centrifugation and washing with PBS to obtain cell pellets, which was subjected to nano-LC/MS/MS, performed at the School of Pharmacy, UW-Madison. Briefly, proteins were extracted from the frozen cell pellets after addition of 0.3 mL ice-cold PBS and passing cells through a 23-gauge needle 10–15 times. Cell lysates were then cleared by centrifugation at 10,000 g for 10 min at 4°C. Protein concentration of the extracts was then determined by Micro BCA (Thermo Fisher Scientific/Pierce). Sample protein (20 *μ*g) was digested with 1 *μ*g sequencing grade trypsin (Promega Corp.). Following an overnight digestion, samples were prepared for LC/MS/MS by C18 Zip-Tip purification according to the manufacturer's protocol (Millipore Inc.). Samples were then suspended in water with 0.1% formic acid (*v*/*v*) and subjected to nano-LC/MS/MS.

For LC/MS/MS, the samples were analyzed by injecting 1 *μ*g of the digest onto a reverse phase BEH C18 column (100 *μ*m × 100 mm), with 1.7 *μ*m and 300 Å pore particle size (Waters Corp. Milford) using a Waters nanoACQUITY chromatography system. Peptides were eluted from the column using a 180 min increasing organic gradient. Solvent A was water/0.1% formic acid (*v*/*v*), while solvent B was acetonitrile/0.1% formic acid (*v*/*v*). The gradient started at 3% B and increased with a linear gradient to 35% B at 130 min. At 140 min, the gradient increased to 95% B and held for 10 min. At 160 min, the gradient returned to 3% to reequilibrate the column for the next injection. Peptides eluting from the column were analyzed by data-dependent MS/MS on a Q-Exactive Orbitrap mass spectrometer (Thermo Fisher Scientific Inc.). A top 15 method was used to acquire data. The instrument settings were as follows: the resolution was set to 70,000, the AGC target was set to 106 counts, the scan range was from 300 to 2000 m/z, the MS scan was recorded in profile while the MS/MS was recorded in centroid mode, dynamic exclusion was set to 25 seconds.

### 2.10. Data Processing and Protein Identification by Human Database Search

Following LC/MS/MS acquisition, the data were searched against the Swiss-Prot human proteome database with decoy using Sequest HT search engine under the Proteome Discoverer 1.4 software (Thermo Fisher Scientific Inc.). Proteins were identified at a false discovery cutoff <1%. Following protein identification, the LC/MS/MS data were aligned using Chromalign software. Quantitation of peptides eluted between 30 and 130 min was performed on the processed data using SIEVE 2.1 (Thermo Fisher Scientific Inc.).

### 2.11. Pathway Analysis

To understand pathways modulated by MEP, a list of differentially expressed (>1.7 fold) was compiled. These proteins were categorized according to their Gene Ontology (GO) descriptions using information from the GO database and PANTHER (Protein ANalysis THrough Evolutionary Relationships; http://www.pantherdb.org/) classification systems. The proteins were projected based on their molecular functions, biological processes, and protein classes. The canonical pathways, disease/function pathways, and protein-protein interactions were analyzed using Ingenuity Pathway Analysis Software (IPA trial version, Ingenuity Systems, http://www.ingenuity.com) (Qiagen). The predicted protein-protein interaction networks and canonical pathways were generated using inputs of gene identifiers, log2 fold-changes and *p* values between control and treated group comparisons.

### 2.12. Ethical Statement for Animal Studies

Athymic nude mouse studies were conducted according to the institutional guidelines for the care and use of animals and were approved by Animal Care and Use Committee, School of Medicine and Public Health, University of Wisconsin-Madison. The ethical approval protocol number for xenograft studies using Athymic nude mouse is IACUC-M005969.

### 2.13. In Vivo Tumor Xenograft Model

Athymic (nu/nu) female nude mice obtained from NxGen Biosciences (San Diego, CA) were housed under pathogen-free conditions (12 h light/12 h dark schedule) and fed with an autoclaved diet *ad libitum*. Tumor xenografts in mice were established by subcutaneous injection of a luciferase tagged PC3M-LUC-C6-luc-6 cells (1.5 × 10^6^) used for bioluminescence studies, mixed with matrigel (Collaborative Biomedical Products, MA), in a ratio of 1 : 1, on both flanks of the animals. Twelve animals were randomly selected into two groups consisting of six animals each. The first group received DMSO while the second group received MEP, 1.25 mg/animal, intra-peritoneally twice weekly. Body weights were recorded weekly throughout the study. Tumor sizes were measured weekly, and tumor volume was calculated by the formula 0.5238 × L × B × H (L = length, B = breadth, and H = height of the tumor).

### 2.14. Bioluminescent Imaging

Mice were injected with d-luciferin (150 mg/kg) and placed onto the warmed stage inside the camera box, continuously exposed to 2% isoflurane to maintain sedation during imaging. *In-vivo* bioluminescent imaging was performed using the IVIS Imaging System (Xenogen) on the last day of the study prior to excision of the tumor. Animals were euthanized by CO_2_ inhalation method following ARAC guidelines, and tumors were removed, snap frozen, or fixed in 10% formalin for further studies. Imaging time ranged from 1 second to 2 minutes, depending on the bioluminescence, and 5 mice were imaged simultaneously.

### 2.15. Statistical Analysis

All statistical analysis was carried out with Graph Pad prism 6 (San Diego, CA), using Student's *t*-test, *p* values < 0.05 were considered statistically significant.

## 3. Results

### 3.1. MEP Exerts a Deleterious Effect on PCa Cell Viability

**Figure 1:**
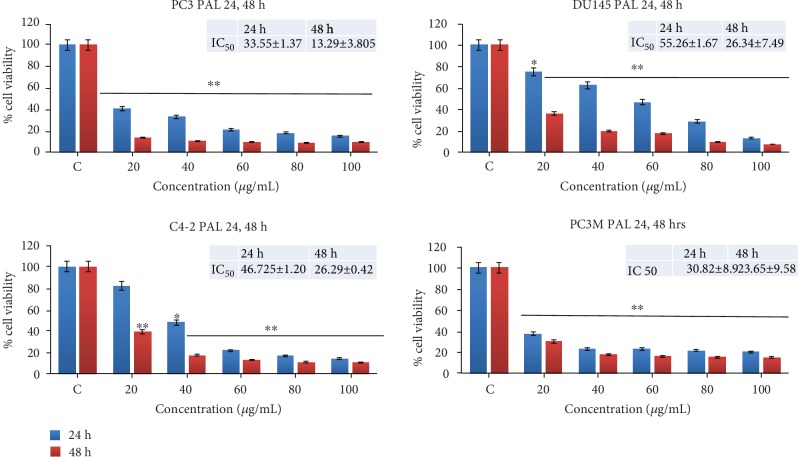
MEP exerts a deleterious effect on PCa cell viability. PC3, DU145, C42, and PC3M at 24 and 48 h. Mean ± SD of experiments performed in triplicate as shown. ^∗^*p* < 0.05 and ^∗∗^*p* < 0.01 were considered statistically significant.

**Figure 2:**
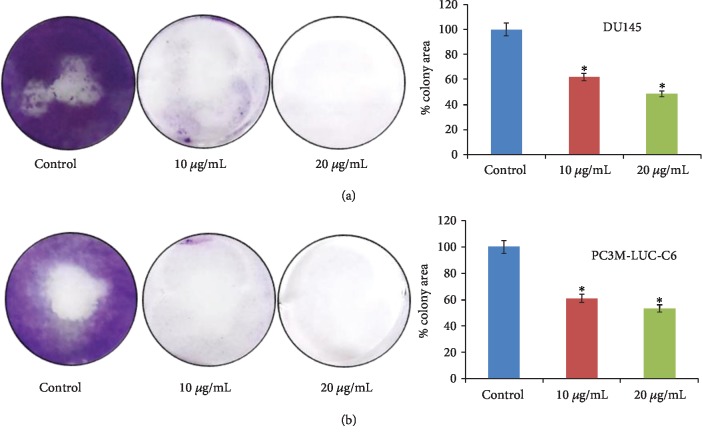
MEP inhibits long-term viability of PCa cells as assessed by the colony formation assay. (a) Effects of MEP on DU145 cells and densitometric analysis of the colony area. (b) Effects of MEP on PC3M cells and densitometric analysis of the colony area. Experiment was performed in triplicate. ^∗^*p* < 0.05 and ^∗∗^*p* < 0.01 were considered statistically significant.

To examine its effect on cell viability, we performed the MTT assay in MEP-treated androgen-independent (PC3, DU-145, and PC3M-LUC-C6) and androgen-sensitive (C4-2) PCa cells. We observed dose-dependent inhibition of growth and viability in MEP treated (10-100 *μ*g/mL for 24 h and 48 h) PCa cells. The cytotoxic effect of MEP was more pronounced at extended time points, with a marked decrease in cell viability at 48 h. As shown in Figure 1, the IC_50_ values of MEP-treated PC3 cells were 33.6 and 13.3 *μ*g/mL; for DU-145, 55.3 and 26.34 *μ*g/mL; for C4-2_,_ 46.7 and 26.3, and for PC3M-LUC-C6, 30.8 and 23.7, at 24 and 48 h, respectively. Clonogenic studies were conducted to determine the long-term effect of MEP treatment on PCa cells. DU-145 and PC3M-LUC-C6 cells treated for seven days with MEP at 10 and 20 *μ*g/mL showed a significant dose-dependent inhibition of colony formation relative to untreated controls (Figure 2).

### 3.2. MEP Induces G1 Phase Arrest in PCa Cells

**Figure 3:**
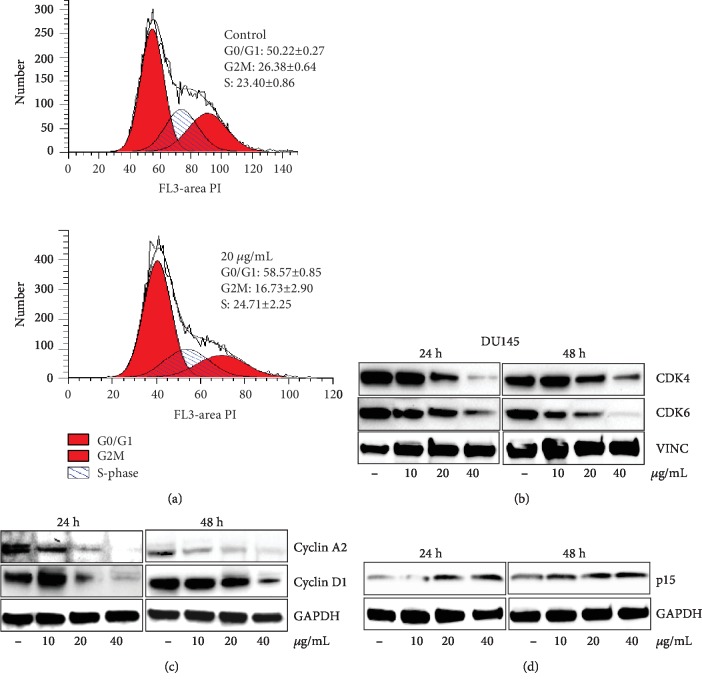
MEP induces G1 phase arrest in PCa cells. (a) Percentage of cell population in each phase of the cell cycle as shown. MEP-treated DU145 cells (20 *μ*g/mL: 24 h). Mean ± SD of experiments performed in triplicate shown. (b–d) Dose-dependent effect of MEP treatment on cell cycle regulatory proteins (10-40 *μ*g/mL: 24 and 48 h). Equal loading was confirmed by reprobing with vinculin or GAPDH.

The effect of MEP treatment on the cell cycle distribution was ascertained using flow cytometry, and the cell cycle profile of MEP-treated PCa cells was generated. The cell cycle distribution for the control was G0/G1: 50.2%, G2/M: 26.4%, and S: 23.4% while at a final concentration of 20 *μ*g/mL, G0/G1: 58.6%, G2/M: 16.7%, and S: 24.7% (Figure 3(a)). Thus, MEP treated cells showed a significant increase in the cell population in the G0/G1 phase at 20 *μ*g/mL when compared with the control. The effect of MEP on cell cycle regulatory proteins was examined via Western blot analysis. Treatment of DU-145 with MEP resulted in decreased protein expressions of Cdks 4 and 6 and cyclins D1 and A2 compared to the untreated controls (Figures 3(b) and 3(c)). This was accompanied with induction of Cdk inhibitor p15 in MEP-treated cells (Figure 3(d)).

### 3.3. MEP Induces Apoptosis via Activation of the Intrinsic Pathway

**Figure 4:**
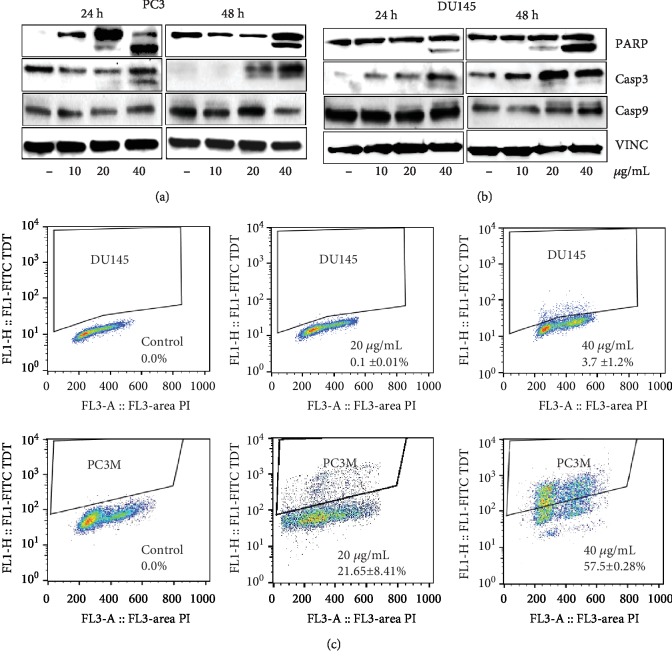
MEP triggers apoptosis through activation of the intrinsic pathway. (a, b) Effects of MEP (10-40 *μ*g/mL: 24 and 48 h) on proteins involved in the apoptotic pathway in PC3 and DU145 cells. Equal loading was confirmed by reprobing with vinculin (c) DU145 and PC3M cells treated with MEP (20 and 40 *μ*g/mL: 24 h) labelled with FITC and analysed by flow cytometry. Percentage of apoptotic cells shown. Mean ± SD of experiments performed in triplicate shown.

To understand if cycle cell arrest is linked to decrease in viability and to ascertain the mechanism of cell death, we evaluated apoptotic markers in MEP-treated cells. MEP-treated cells showed a dose-dependent increase in the expression of cleaved caspase 3 accompanied with PARP cleavage (Figure 4(b)); PC3M-LUC-C6 and DU-145 cells, treated with MEP (20 and 40 *μ*g/mL) were next subjected to flow cytometric analysis in order to quantify the number of cells undergoing apoptosis. We observed a significant dose-dependent increase in the population of apoptotic cells in both cell lines. The apoptotic cells quantified for MEP-treated PC3 cells were 0.0%, 21.7%, and 57.5% and for MEP-treated DU-145 cells were 0.0%, 0.1%, and 3.7% at 0, 20, and 40 *μ*g/mL, respectively (Figure 4(b)). After establishing that apoptosis is the primary mechanism of MEP-induced cell death, we asked if apoptosis is mediated through activation of intrinsic and/or extrinsic pathways. We evaluated the expression of caspases 8 and 9 in MEP-treated PCa cells. We observed that caspase 9 cleavage was enhanced in a dose-dependent manner (Figure 4(a)) while caspase 8 remained unaffected (data not shown). This suggested that MEP induced apoptosis through activation of the intrinsic apoptotic pathway in MEP-treated cells.

### 3.4. MEP Modulates the Proteome of PCa Cells

**Figure 5:**
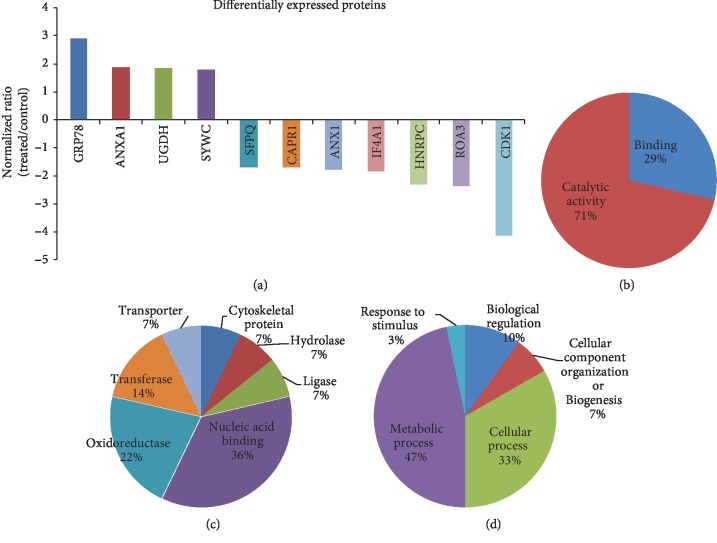
MEP modulates the proteome of PCa cells. (a) Proteins showing >1.7-fold change in abundance with MEP treatment (95% confidence interval and *p* value). Gene Ontology analysis of proteome changes. Identified proteins showing ≥1.7-fold change were systematized based on (b) molecular function, (c) protein classes, and (d) biological process by the PANTHER classification system.

**Figure 6:**
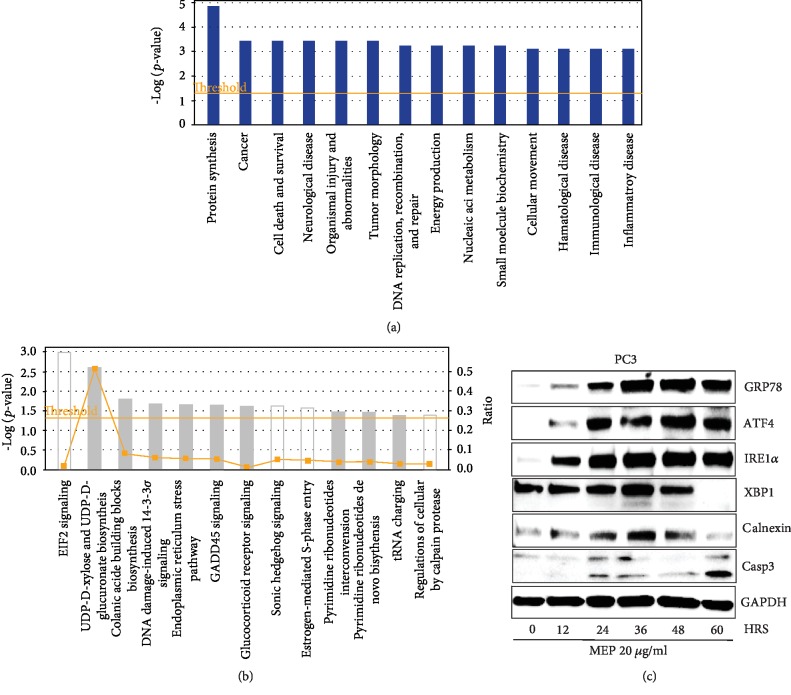
MEP-induced apoptosis is associated with the activation of the ER stress pathway. (a) IPA was used to classify the proteins on the basis of disease and functional relation to altered proteins. (b) Top canonical pathways significantly altered upon MEP treatment. (c) Western blot validation of GRP78, ER stress proteins, and caspase 3 in a time-dependent study on MEP-treated (20 *μ*g/mL) PC3 cells. Equal loading was confirmed by reprobing with GAPDH.

To examine the effect of MEP on the proteome of PCa cells, we treated PC3M-LUC-C6 cells with MEP (20 *μ*g/mL: 24 h) and conducted a LC/MS/MS analysis. This analysis resulted in the identification of a total of 1012 proteins with a 0.05 confidence interval (CI). These proteins were then further screened based on fold change. We selected 11 proteins showing statistically significant (*p* < 0.05) change ≥1.7-fold (Table 1) for further analysis. Details of these proteins including their protein ID, protein name, number of unique peptides, hits (MS/MS identification scans), and fold change upon MEP treatment are presented in Suppl. [Supplementary-material supplementary-material-1]. The fold change increase or decrease in MEP-modulated proteins are represented in Figures 5 and 6.

The selected proteins annotated with the GO terms were initially examined for their biological functions using the PANTHER classification system. A scheme showing the distribution of proteins among molecular functions, biological processes, and protein classes is shown in Figures 5(b)–5(d), respectively. Most of the differentially regulated proteins, when classified based on molecular functions, were found to have catalytic activity (71%) while the rest (29%) were associated with binding (Figure 5(b)). Moreover, MEP affected a broad category of proteins, namely, nucleic acid binding, oxidoreductases, transporters, transferases, ligases, hydrolases, and cytoskeletal proteins (Figure 5(c)). The GO analysis categorizing proteins pertinent to biological processes demonstrated major involvement in metabolic and cellular processes (Figure 5(d)). MEP also affected biological regulation, developmental process, localization, cellular component organization, etc. In conclusion, the analysis indicated that MEP affects growth, proliferation, and several other critical cellular processes.

Next, canonical pathway analysis, putative networks, and protein-protein relationships of the differentially expressed proteins were elucidated using the IPA software. The selected proteins (Table 1) were plugged into the IPA module with their corresponding Swiss-Prot IDs and respective fold changes to map proteins into biological networks and identify key functional pathways. Thirteen canonical pathways linked to MEP-modulated proteins were identified, including EIF2 signaling and the endoplasmic reticulum stress pathway (Figure 6(b)). We further integrated MEP-regulated proteins into distinct interaction networks, to predict the involvement of disease and function-related processes. As shown in Figure 6, most of these networks account for biological functions related to cancer, cell death and survival, tumor morphology, DNA replication, recombination, and repair.

### 3.5. MEP-Induced Apoptosis Is Associated with Activation of the ER Stress Pathway

Next, we attempted to validate the data obtained from PANTHER and IPA analysis. We focused our attention on the ER stress pathway significantly modulated in MEP-treated PCa cells. Western blot analysis of GRP78/BiP, a central regulator for ER stress, demonstrated significant induction of protein expression in response to MEP treatment. To determine if MEP-mediated apoptosis is linked to activation of the ER stress pathway, we conducted time course studies where PC3M-LUC-C6-luc cells treated for different time points with MEP were evaluated for components of the ER stress pathway and apoptotic marker caspase 3. We noted induction of BiP and other key players including ATF4 and IRE1*α* as early as 12 h posttreatment. The splicing of XBP1 was evident at 24 h, but was more pronounced at 36 h, after which protein expression was lost at 60 h. A similar trend was noted with calnexin, which returned to basal levels at the longer time point. Notably, cleavage of caspase 3 was induced at 24 h; however, significant increase in cleaved caspase 3 expression was observed at 60 h (Figure 6). Taken together, the data indicated that ER stress preceded apoptotic events in MEP-treated cells.

### 3.6. MEP Inhibits the Growth of Metastatic PC3M-LUC-C6 Tumors in Athymic Nude Mice

**Figure 7:**
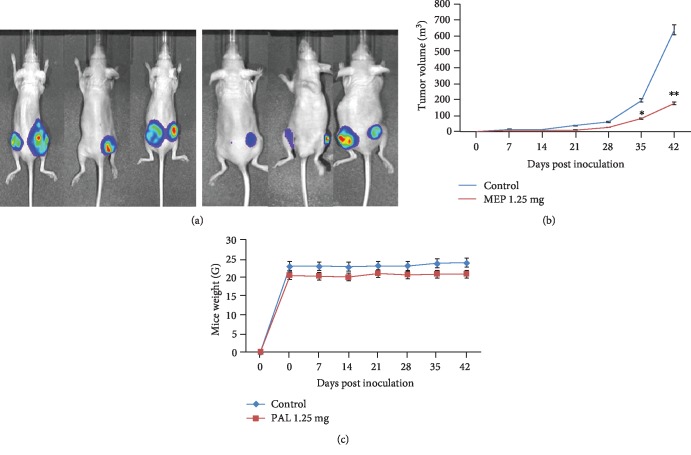
MEP inhibits the growth of metastatic PC3M-LUC-C6 tumors in athymic nude mice. (a) Representative bioluminescence of PC3M-luc-6 tumor-bearing mice after 6 weeks of treatment. (b) Line graph showing tumor growth determined by weekly measurements of the tumor volume. (c) Line graph showing the animal weight. Each value in the graph is the mean ± SD from 6 mice. ^∗^*p* < 0.05 and ^∗∗^*p* < 0.01 were considered statistically significant.

To monitor the effect of MEP on the growth of PCa in an *in vivo* setting, PC3M-LUC-C6-luc-6 cells were implanted subcutaneously in 12 athymic nude mice. Two weeks after implantation, mice were randomly divided into two groups and administered MEP or DMSO intraperitoneally for 6 weeks. No apparent toxicity was noted in treated mice using body weight as an observational parameter. MEP treatment significantly (*p* = 0.001) inhibited tumor growth compared to control (Figure 7(a)). The control group showed a 46-fold increase in the tumor volume over 6 weeks. In contrast, only a 20-fold increase was evident in mice receiving MEP. This translated to a 73% inhibition in tumor growth in MEP-treated animals. Reduction in tumor volumes in MEM-treated animals was further validated through bioluminescent imaging of animals injected with D-luciferin (Figure 7(b)).

### 3.7. The Active Constituent Tetranorditerpene Isolated from MEP Induces Apoptosis in PCa Cells

**Figure 8:**
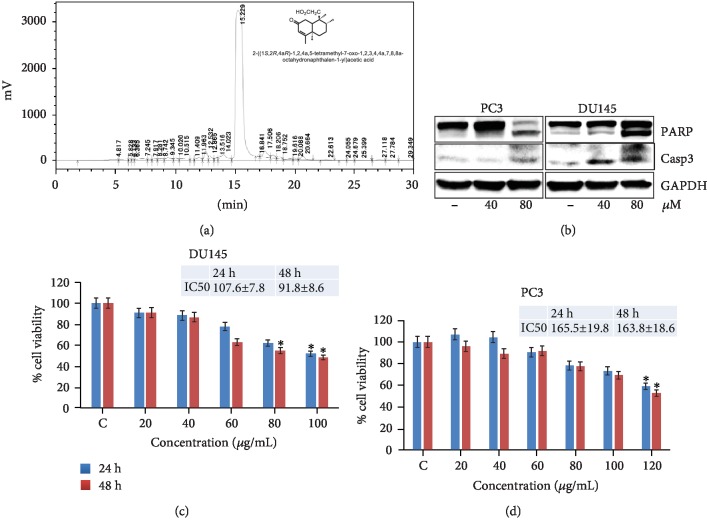
The active constituent tetranorditerpene isolated from MEP induces apoptosis in PCa cells (a) Isolation of tetranorditerpene from MEP using HPLC. (b) Dose-dependent effect of isolated tetranorditerpene on PARP and caspase 3; equal loading was confirmed by GAPDH. (c, d) Cell viability assay against DU145 and PC3 cells as shown by their IC_50_. Mean ± SD of experiments performed in triplicate shown. ^∗^*p* < 0.05 and ^∗∗^*p* < 0.01 were considered statistically significant.

We had previously reported the purification and isolation of a rare tetranorditerpene from MEP that we characterized using NMR spectroscopic analysis (Figure 8(a)). The compound was confirmed to be a tetranorditerpene 1-naphthalene acetic-7-oxo-1,2,3,4,4a,7,8,8a-octahydro-1,2,4a,5-tetramethyl acid [29]. To ascertain if this is the major active cytotoxic principle from the extract, we performed MTT assay on PCa cells treated with the isolated compound. The IC_50_ values at 24 and 48 h were calculated as 106.6 and 91.8 *μ*M for DU145 and 165.5 and 163.8 *μ*M for PC3, respectively, as depicted in Figures 8(c) and 8(d). We then treated PCa cells and evaluated the effect of the compound on the apoptotic pathway. Immunoblot analysis showed marked cleavage of PARP and caspase 3 at 80 *μ*M (Figure 8(b)) indicating that the compound may be partially responsible for the observed growth inhibitory effects of MEP on PCa cells.

## 4. Discussion

The use of herbs and plant-based formulations remains the mainstay of therapy in several developing nations [35]. Medicinal plants use is also widely accepted in many developed countries with complementary and alternative medicine becoming established in Europe, Australia, and North America [36]. However, there are several challenges associated with the field of traditional medicines including incorrect usage, inadequate quality control, and lack of proper scientific justification and information [37]. Many of these challenges have been overcome with increasing focus on toxicological and efficacy studies *in vitro* and *in vivo*. Strategies and polices are also being fostered by WHO-AFRO to update databases on medicinal plants through the provision of guidelines for documentation of herbal formulas [38].

*Polyalthia longifolia* has been used for the management of gastric ulcer, hypertension, inflammation, microbial infections, etc. We recently isolated active principles from the plant including clerodane diterpene, tetranorditerpene, and other derivatives. We further showed that both tetranorditerpene and clerodane diterpene exerted cytotoxic effect on HL-60 leukemic cell lines [28, 29]. Clerodane diterpene possessed drug resistance modifying potential against methicillin-resistant strain of *Staphylococcus aureus* [39]. *Polyalthia longifolia* methanol leaf extract has also been shown to induce apoptosis and cell cycle arrest in the HeLA cervical cell line [26]. In the current study, we examined the efficacy of MEP against PCa and determined the molecular basis of its observed effects.

A critical aspect of cancer development is a dysregulated cell cycle and aberrant DNA replication leading to clonal expansion, proliferation, and accumulation of the tumor mass [40]. The progression of a cell cycle is coordinated by a family of protein kinase complexes, comprised of a catalytic subunit, the cyclin-dependent kinase, and its activating partner—cyclin [41]. Inhibitors of cyclin D1/Cdks 4/6 complexes have been explored as therapeutic targets as this checkpoint was found deregulated in several human cancers [42]. Analysis of cell viability in short-term and cell proliferation in long-term studies showed that MEP is a potent inhibitor of PCa cell proliferation. Cell cycle studies substantiated this observation, as MEP-treated PCa cells were arrested specifically at the G1 checkpoint with the accumulation of cell cycle inhibitor p15 and reduction in Cdks 4/6 and cyclins A and D expression. Apoptosis, characterized by cleavage of chromatin and distinctive morphologic changes in the nucleus and cytoplasm, serves as a balance to mitosis in the regulation of cell growth. A number of anticancer strategies have successfully aimed at targeting the apoptotic machinery to induce tumor cell death [43]. Employing several PCA cell lines, we established that MEP-mediated cell cycle arrest was associated with the induction of apoptosis in PCa cells. Moreover, MEP-induced apoptosis resulted from the activation of the intrinsic apoptotic pathway, as evidenced by cleavage of caspases 3 and 9, with no effect of the extract on caspase 8 signaling.

The recent discovery that GRP78/BiP is present on the cell surface of tumors but not in normal organs represents an exciting opportunity for using it as a biomarker and/or targeting its function to harness cell growth [44]. Remarkably, this ER resident chaperone with a critical role in oncogenic stress was the most significantly modulated protein in MEP-treated cells. Consistent with its role as an ER stress signaling regulator, BiP facilitates proper protein folding and targets misfolded proteins for proteasomal degradation [45]. Under normal conditions, the luminal domains of PERK and ATF6 proteins are bound to BiP, keeping them in an inactive state. However, under stress, BiP is released from these complexes. In contrast to PERK and ATF6, which are modulated by association with BiP, IRE1*α* is activated when unfolded proteins bind directly to it [46]. Our studies show that the activation of BiP occurred as early as 12 h post MEP treatment. Induction of IRE1*α* in MEP-treated cells indicates that parallel inputs may be involved in activating the two arms of ER stress signaling. Upon activation, IRE1*α* catalyzes the excision of a 26-nucleotide intron from the XBP1 mRNA causing a frame shift in the XBP1 coding sequence and translation of the spliced XBP-1 isoform [47]. Activation of IRE1*α* was associated with increase in the spliced XBP1 variant in MEP-treated cells. Our data indicates that the accumulation of misfolded proteins far exceeding its fold capacity leads to ER stress, stimulation of eIF2 signaling, and cell cycle arrest in MEP-treated cells. Increase in ATF4 levels indicated that apoptosis in MEP-treated cells might well be mediated through ATF4-CHOP induction of proapoptotic genes and/or suppression of antiapoptotic Bcl-2 proteins.

After delineating key targets of MEP in *in vitro* studies, we determined the efficacy of MEP in an *in vivo* PCa model. The data from xenograft studies validated the growth inhibitory potential of MEP, evident from a significant reduction in the tumor volume. We assessed tumor growth not only through evaluation of the tumor size at the end of the study but also confirmed it using bioluminescence imaging. Notably, no signs of toxicity were observed in the extract-treated group. Nonetheless, detailed follow-up studies with toxicological workup are warranted to substantiate our preliminary findings.

Finally, it is important to identify the active principles in the plant extract that are driving the desired effect. Studies have focused on the fractionation and isolation of plant-based extracts with the intent of identifying and retrieving their active principles for further development and analysis. However, it was observed that many of these isolated compounds do not have activity comparable with the plant extracts from which they were isolated [48]. We had previously reported the isolation of a rare tetranorditerpene from MEP and showed its effects on human leukemic HL-60 cells [29]. To determine if this compound was primarily responsible for the growth inhibitory effect of MEP, we evaluated its effect on PCa cell proliferation. We observed reduced cell viability and induction of apoptosis; however, this effect was observed at relatively higher doses. Our data suggest a potential synergy between macro and micro constituents present in MEP, which may be responsible for the potent activity of the extract and need to be explored in detail. In conclusion, our findings provide preliminary evidence of the efficacy of MEP against PCa in *in vitro* and *in vivo* models. Further studies are required to evaluate its activity in other preclinical models of cancer for its translation as a potent anticancer agent.

## Figures and Tables

**Table 1:** Details of upregulated and downregulated proteins with >1.7-fold change in abundance with MEP treatment (95% confidence interval and *p* value).

Protein ID	Gene name	Protein description	Fold change
P11021	GRP78	Glucose-regulated protein	2.9
P04083	ANXA1	Annexin A1	1.9
O60701	UGDH	UDP glucose 6 dehydrogenase	1.8
P23381	SYWC	Tryptophan tRNA ligase cytoplasmic	1.8
P23246	SFPQ	Splicing factor proline and glutamine rich	-1.7
Q14444	CAPR1	Caprin-1	-1.7
P50995	ANX11	Annexin A11	-1.8
P60842	IF4A1	Eukaryotic initiation factor 4A-I	-1.8
P07910	HNRPC	Heterogeneous nuclear ribonucleoproteins C1/C2	-2.3
P51991	ROA3	Heterogeneous nuclear ribonucleoprotein A3	-2.4
P06493	CDK1	Cyclin-dependent kinase 1	-4.1

## Data Availability

The data used to support the findings of this study are available from the corresponding author upon request.

## Abbreviations

ARAC: Animal Research Advisory Committee
ATF4: Activating transcription factor 4
BIP: Binding immunoglobulin protein
CDK: Cyclin-dependent kinase
ERAD: ER-associated protein degradation
ER Stress: Endoplasmic reticulum stress
GO: Gene Ontology
GRP78: Glucose-regulated protein 78
IPA: Ingenuity pathway analysis
IRE*α*1: Inositol-requiring enzyme-1*α*
MEP: Methanol extract of *Polyalthia longifolia*
PANTHER: Protein analysis through evolutionary relationship
PSA: Prostate-specific antigen
UPR: Unfolded protein response
XBP1: X-box binding protein 1.

## Supplementary Materials

Supplementary Materials Figure S1: MEP Induces G1 phase Arrest in PCa Cells: Densitometric analysis of Cdk4 and Cdk6 expression in MEP treated cells. Whole cell lysates of DU-145 PCa cells, treated with MEP (10-40*μ*M:24&48 hrs.) were analyzed for Cdk4 and Cdk6 protein expression. Equal loading was confirmed by reprobing for Vinculin. Relative density of the bands were computed using the Imagej software normalized to Vinculin. Figure S2: MEP Induces G1 phase Arrest in PCa Cells: Densitometric analysis of Cyclin A2 and Cyclin D1 expression in MEP treated cells. Whole cell lysates of DU-145 PCa cells, treated with MEP (10-40*μ*M:24&48 hrs.) were analyzed for Cyclin A2 and Cyclin D1 protein expression. Equal loading was confirmed by reprobing for Vinculin. Relative density of the bands were computed using the Imagej software normalized to Vinculin. Figure S3: A and B: MEP Induces G1 phase Arrest in PCa Cells: Densitometric analysis of Cdk inhibitor p15 expression in MEP treated cells. Whole cell lysates of DU-145 PCa cells, treated with MEP (10-40*μ*M:24&48 hrs.) were analyzed for p15 protein expression. Equal loading was confirmed by reprobing for Vinculin. Relative density of the bands were computed using the Imagej software normalized to Vinculin. C and D: MEP induces apoptosis in PCa Cells: Densitometric analysis of PARP in MEP treated cells. Whole cell lysates of PC3 PCa cells, treated with MEP (10-40*μ*M:24&48 hrs.) were analyzed for PARP protein expression. Equal loading was confirmed by reprobing forVinculin. Relative density of the bands were computed using the Imagej software normalized to Vinculin. Figure S4: MEP induces apoptosis via the intrinsic apoptotic pathway in PCa Cells: Densitometric analysis of Caspase 3 and 9 in MEP treated cells. Whole cell lysates of PC3 PCa cells, treated with MEP (10-40*μ*M:24&48 hrs.) were analyzed for Caspase 3 and 9 protein expression. Equal loading was confirmed by reprobing for Vinculin. Relative density of the bands were computed using the Imagej software normalized to Vinculin. Figure S5: MEP induces apoptosis via the intrinsic apoptotic pathway in PCa Cells: Densitometric analysis of PARP and Caspase 3 in MEP treated cells. Whole cell lysates of DU145 PCa cells, treated with MEP (10-40*μ*M:24&48 hrs.) were analyzed for PARP and Caspase 3 protein expression. Equal loading was confirmed by reprobing for Vinculin. Relative density of the bands were computed using the Imagej software normalized to Vinculin. Figure S6: MEP induces apoptosis via the intrinsic apoptotic pathway in PCa Cells: Densitometric analysis of Caspase 9 in MEP treated cells. Whole cell lysates of DU145 PCa cells, treated with MEP (10-40*μ*M:24&48 hrs.) were analyzed for Caspase 9 protein expression. Equal loading was confirmed by reprobing for Vinculin. Relative density of the bands were computed using the Imagej software normalized to Vinculin. Figure S7: MEP activates ER stress pathway in PCa Cells: Densitometric analysis of GRP78, ATF4, IRE1*α* and XBP1 in MEP treated cells. Whole cell lysates of PC3 PCa cells, treated with MEP (20*μ*g/mL: 0-60 hr.) were analyzed for GRP78, ATF4, IRE1*α* and XBP1 protein expression. Equal loading was confirmed by reprobing for GAPDH. Relative density of the bands were computed using the Imagej software normalized to GAPDH. Figure S8: MEP activates ER stress pathway associated with apoptosis in PCa Cells: Densitometric analysis of Calnexin and Caspase 3 in MEP treated cells. Whole cell lysates of PC3 PCa cells, treated with MEP (20*μ*g/mL: 0-60 hr.) were analyzed for Calnexin and Caspase 3 protein expression. Equal loading was confirmed by reprobing for GAPDH. Relative density of the bands were computed using the Imagej software normalized to GAPDH. Figure S9: Original western blots showing the concentration-dependent expression of CDK 4, 6; Cyclin A2 and Vinculin upon treatment with MEP (PAL) on PC3 and DU145 cells. Figure S10: Original western blots showing the concentration-dependent expression of Cyclin D1 and time dependent expressions of IRE1 and ATF4 upon treatment with MEP (PAL) on PC3 and DU145 cells. Figure S11: Original western blots showing the concentration-dependent expression of Caspase 3 and Caspase 9 upon treatment with MEP (PAL) on PC3. Figure S12: Original western blots showing the concentration-dependent expression of PARP and time-dependent expressions of XBP1, Calnexin, GRP78 and caspase 3 upon treatment with MEP (PAL) on PC3 cells.
