# Correction to “*Polyalthia longifolia* Extract Triggers ER Stress in Prostate Cancer Cells Concomitant with Induction of Apoptosis: Insights from *In Vitro* and *In Vivo* Studies”

**DOI:** 10.1155/omcl/9757940

**Published:** 2026-04-12

S.O. Afolabi, O.E. Olorundare, A. Babatunde, et al., “*Polyalthia longifolia* Extract Triggers ER Stress in Prostate Cancer Cells Concomitant with Induction of Apoptosis: Insights from *In Vitro* and *In Vivo* Studies” *Oxidative Medicine and Cellular Longevity*, vol. 2019 (2019). https://doi.org/10.1155/2019/6726312.

Errors were reported in Figures 3 and 4. More specifically, the loading controls for the Western Blot experiments presented in these figures were incorrectly placed and labelled. These errors were introduced during figure assembly and should be corrected as follows:

Figure 3MEP induces G1 phase arrest in PCa cells. (a) Percentage of cell population in each phase of the cell cycle as shown. MEP‐treated DU145 cells (20 μg/mL: 24 h). Mean ± SD of experiments performed in triplicate shown. (b–d) Dose‐dependent effect of MEP treatment on cell cycle regulatory proteins (10–40 μg/mL: 24 and 48 h). Equal loading was confirmed by reprobing with vinculin.

**Figure:**
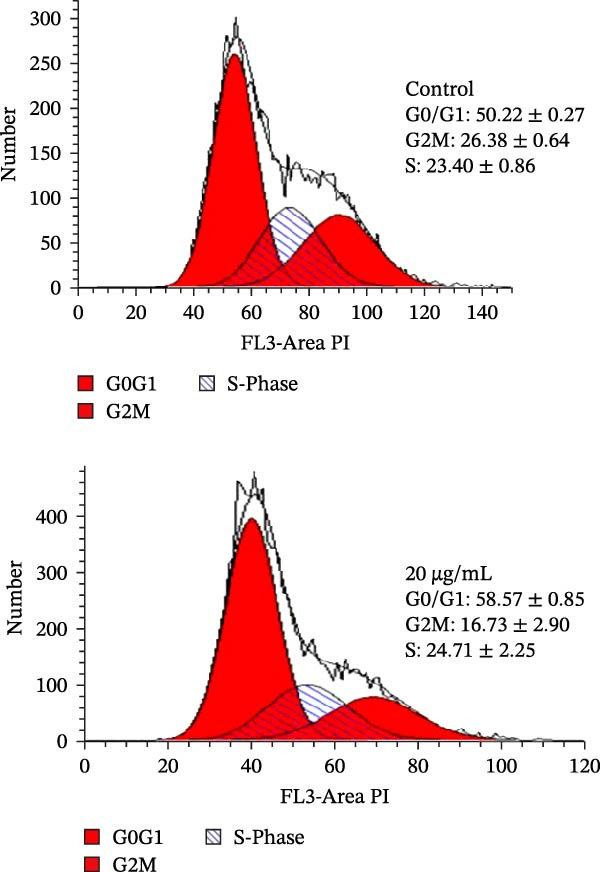
(a)

**Figure:**
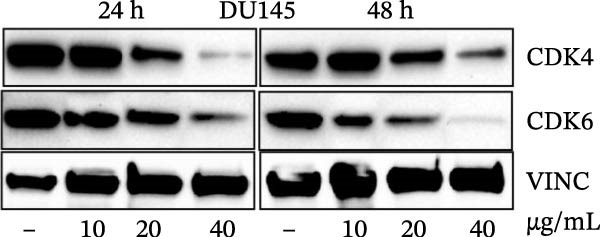
(b)

**Figure:**
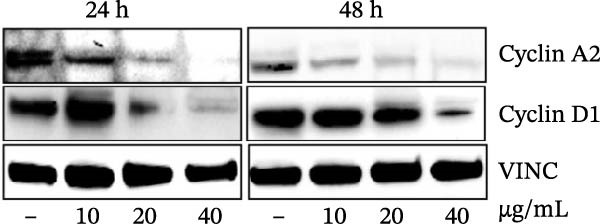
(c)

**Figure:**
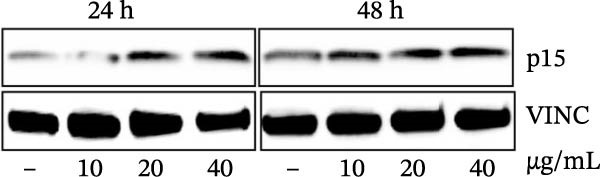
(d)

Figure 4MEP triggers apoptosis through activation of the intrinsic pathway. (a, b) Effects of MEP (10–40 μg/mL: 24 and 48 h) on proteins involved in the apoptotic pathway in PC3 and DU145 cells. Equal loading was confirmed by reprobing with vinculin (c) DU145 and PC3M cells treated with MEP (20 and 40 μg/mL: 24 h) labelled with FITC and analysed by flow cytometry. Percentage of apoptotic cells shown. Mean ± SD of experiments performed in triplicate shown.

**Figure:**
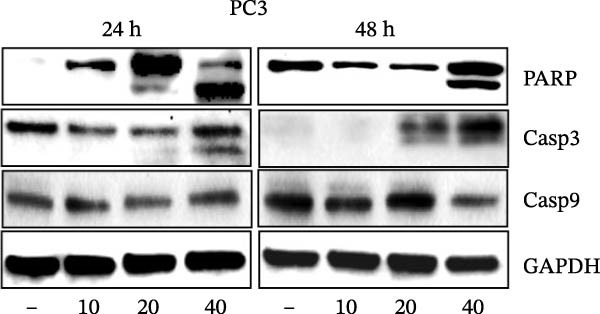
(a)

**Figure:**
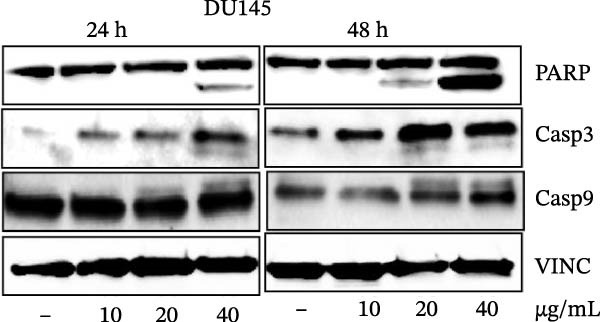
(b)

**Figure:**
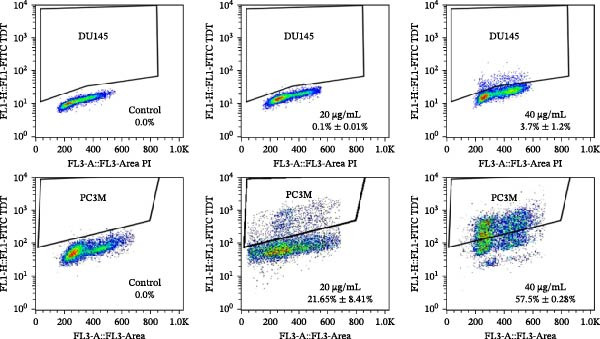
(c)

We apologize for these errors.

