# Supplementary material for: Polyalthia longifolia Extract Triggers ER Stress in Prostate Cancer Cells Concomitant with Induction of Apoptosis: Insights from In Vitro and In Vivo Studies
Source: Oxid Med Cell Longev. 2019 Nov 13;2019:6726312. doi: 10.1155/2019/6726312 (PMC6881593; doi:10.1155/2019/6726312)
Supplement: Supplementary Materials — Figure S1: MEP Induces G1 phase Arrest in PCa Cells: Densitometric analysis of Cdk4 and Cdk6 expression in MEP treated cells. Whole cell lysates of DU-145 PCa cells, treated with MEP (10-40μM:24&48 hrs.) were analyzed for Cdk4 and Cdk6 protein expression. Equal loading was confirmed by reprobing for Vinculin. Relative density of the bands were computed using the Imagej software normalized to Vinculin. Figure S2: MEP Induces G1 phase Arrest in PCa Cells: Densitometric analysis of Cyclin A2 and Cyclin D1 expression in MEP treated cells. Whole cell lysates of DU-145 PCa cells, treated with MEP (10-40μM:24&48 hrs.) were analyzed for Cyclin A2 and Cyclin D1 protein expression. Equal loading was confirmed by reprobing for Vinculin. Relative density of the bands were computed using the Imagej software normalized to Vinculin. Figure S3: A and B: MEP Induces G1 phase Arrest in PCa Cells: Densitometric analysis of Cdk inhibitor p15 expression in MEP treated cells. Whole cell lysates of DU-145 PCa cells, treated with MEP (10-40μM:24&48 hrs.) were analyzed for p15 protein expression. Equal loading was confirmed by reprobing for Vinculin. Relative density of the bands were computed using the Imagej software normalized to Vinculin. C and D: MEP induces apoptosis in PCa Cells: Densitometric analysis of PARP in MEP treated cells. Whole cell lysates of PC3 PCa cells, treated with MEP (10-40μM:24&48 hrs.) were analyzed for PARP protein expression. Equal loading was confirmed by reprobing forVinculin. Relative density of the bands were computed using the Imagej software normalized to Vinculin. Figure S4: MEP induces apoptosis via the intrinsic apoptotic pathway in PCa Cells: Densitometric analysis of Caspase 3 and 9 in MEP treated cells. Whole cell lysates of PC3 PCa cells, treated with MEP (10-40μM:24&48 hrs.) were analyzed for Caspase 3 and 9 protein expression. Equal loading was confirmed by reprobing for Vinculin. Relative density of the bands were computed using the Imagej softwa [file 6726312.f1.pptx]

## Slide 1
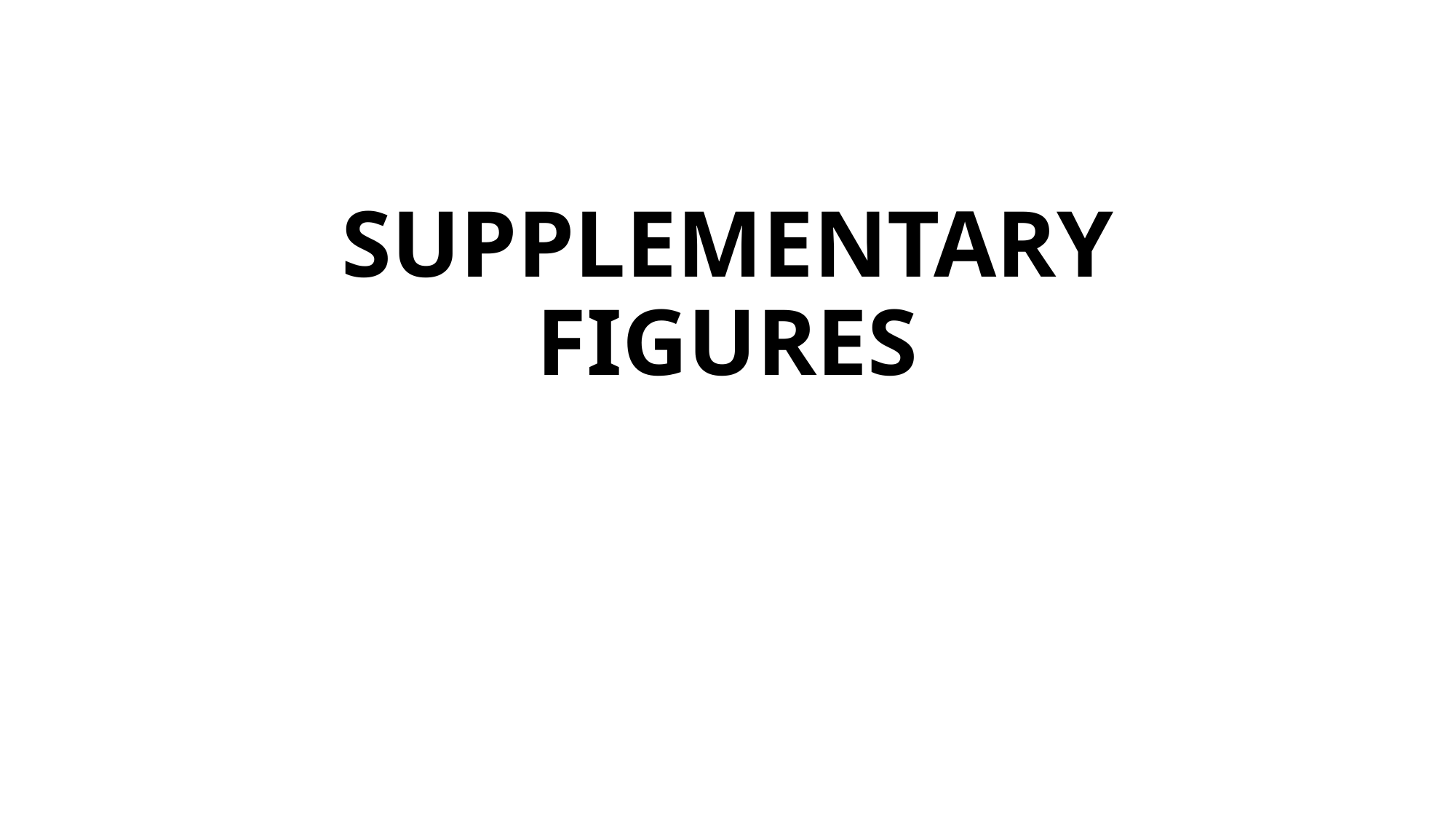

# SUPPLEMENTARY FIGURES

## Slide 2
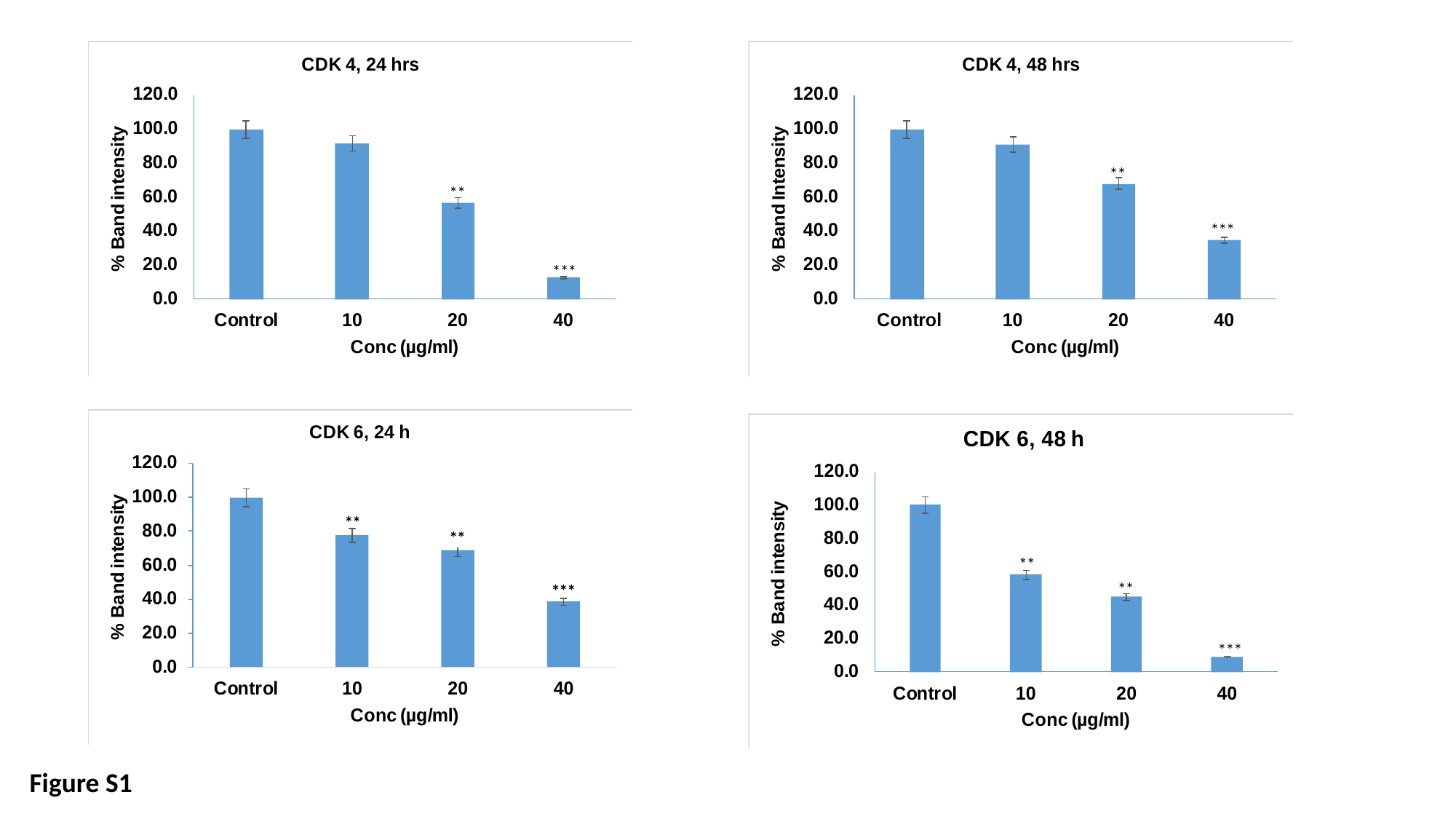

Figure S1

## Slide 3
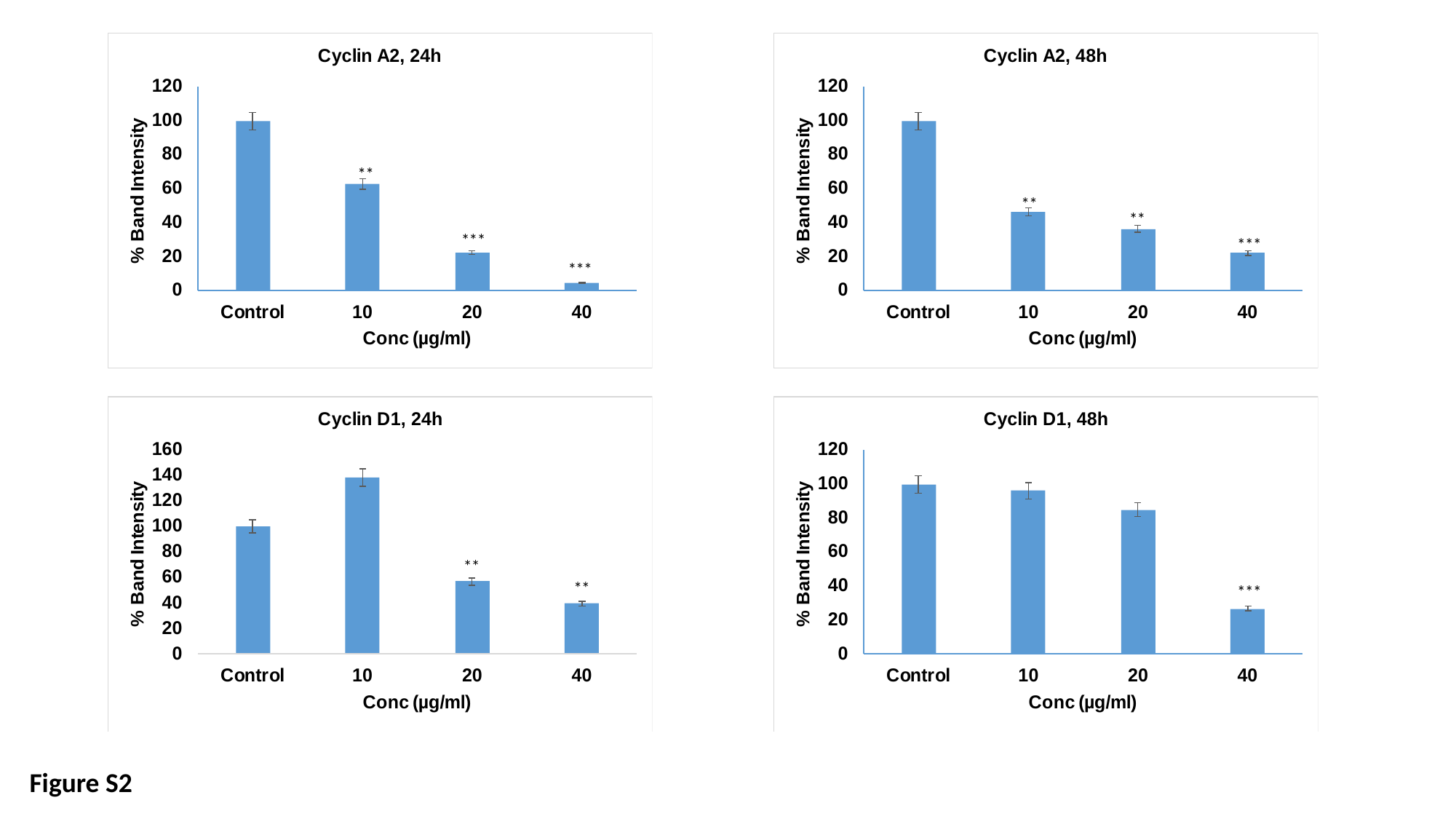

Figure S2

## Slide 4
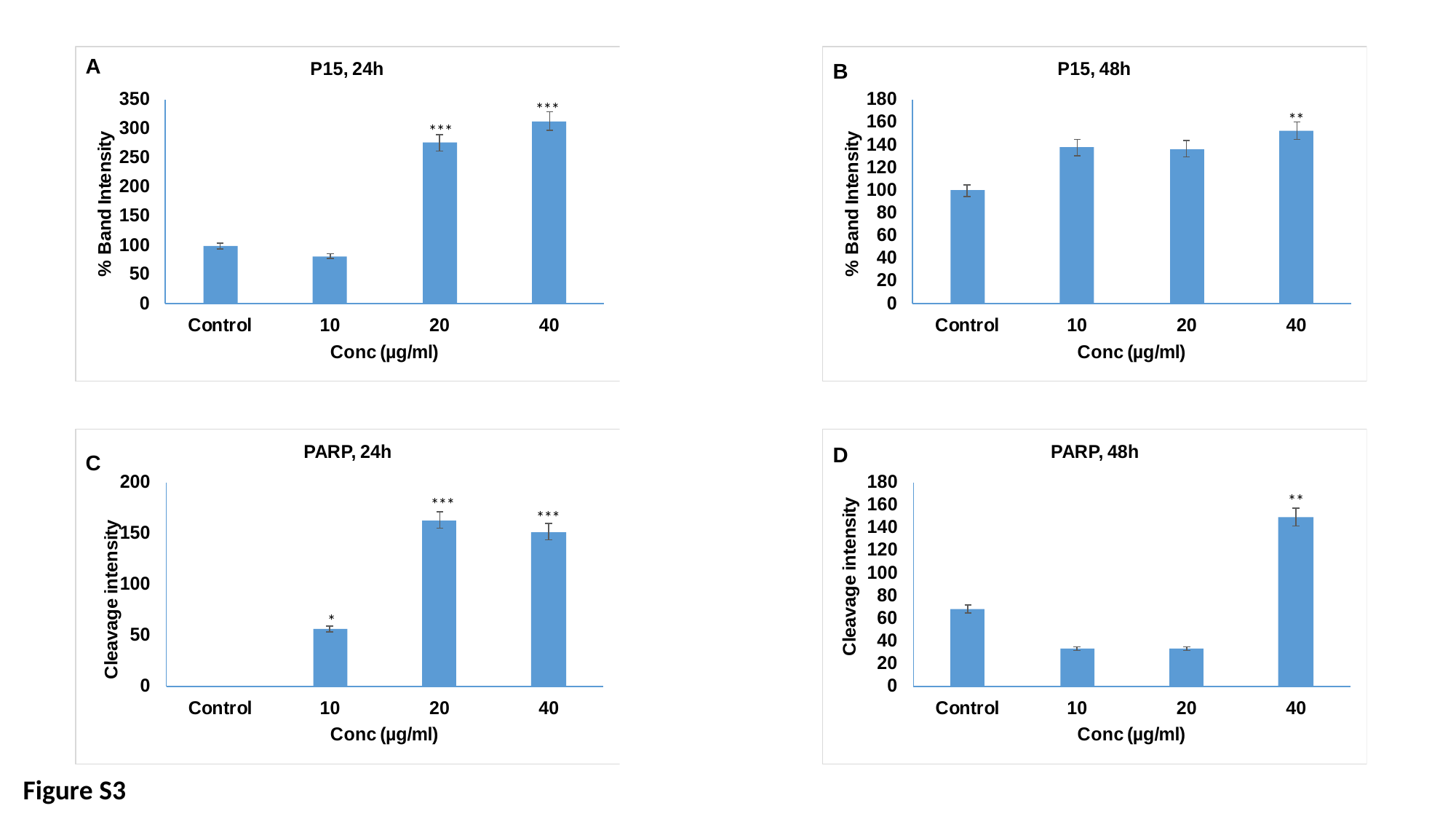

A
B
D
C
Figure S3

## Slide 5
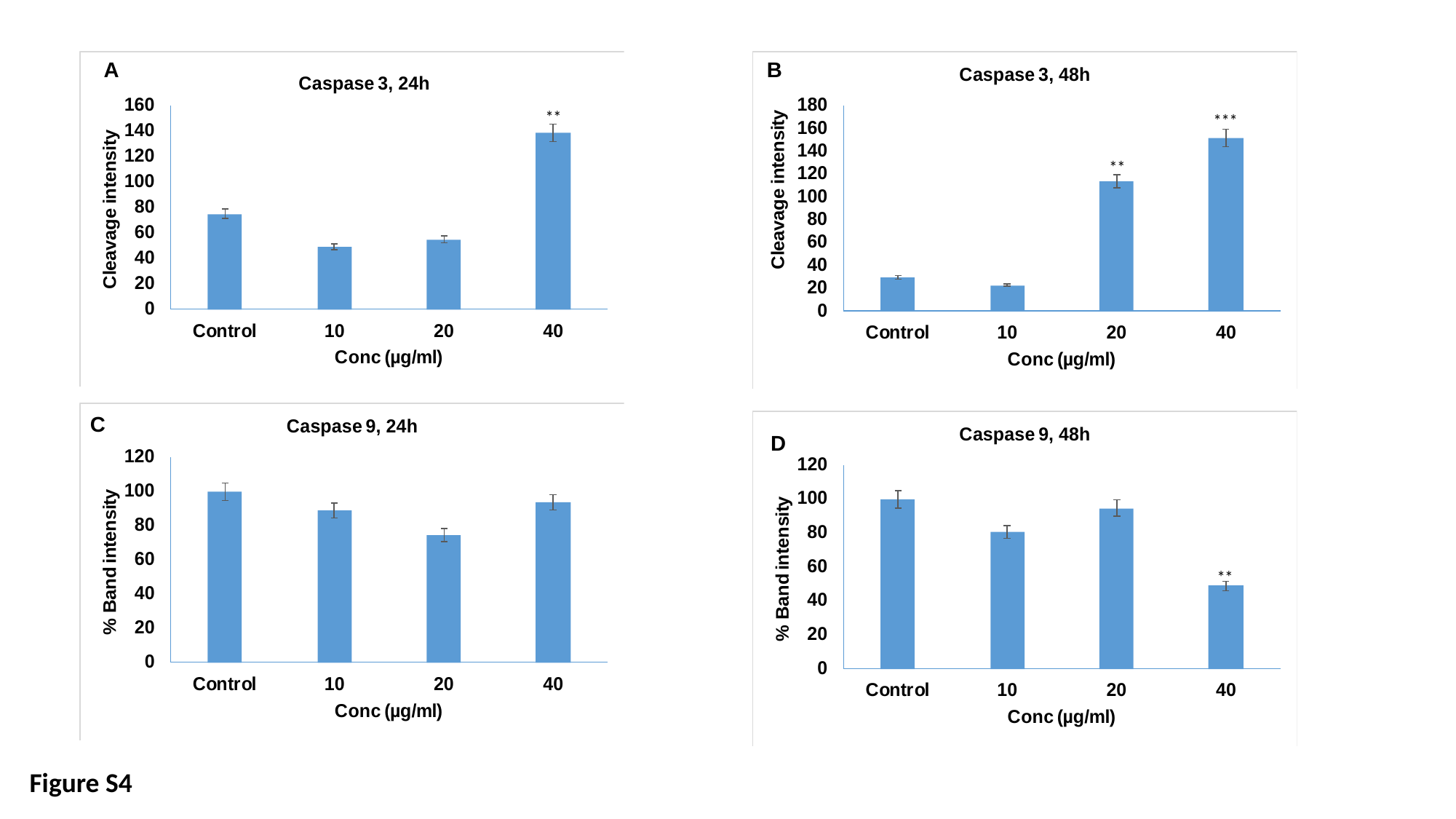

A
B
C
D
Figure S4

## Slide 6
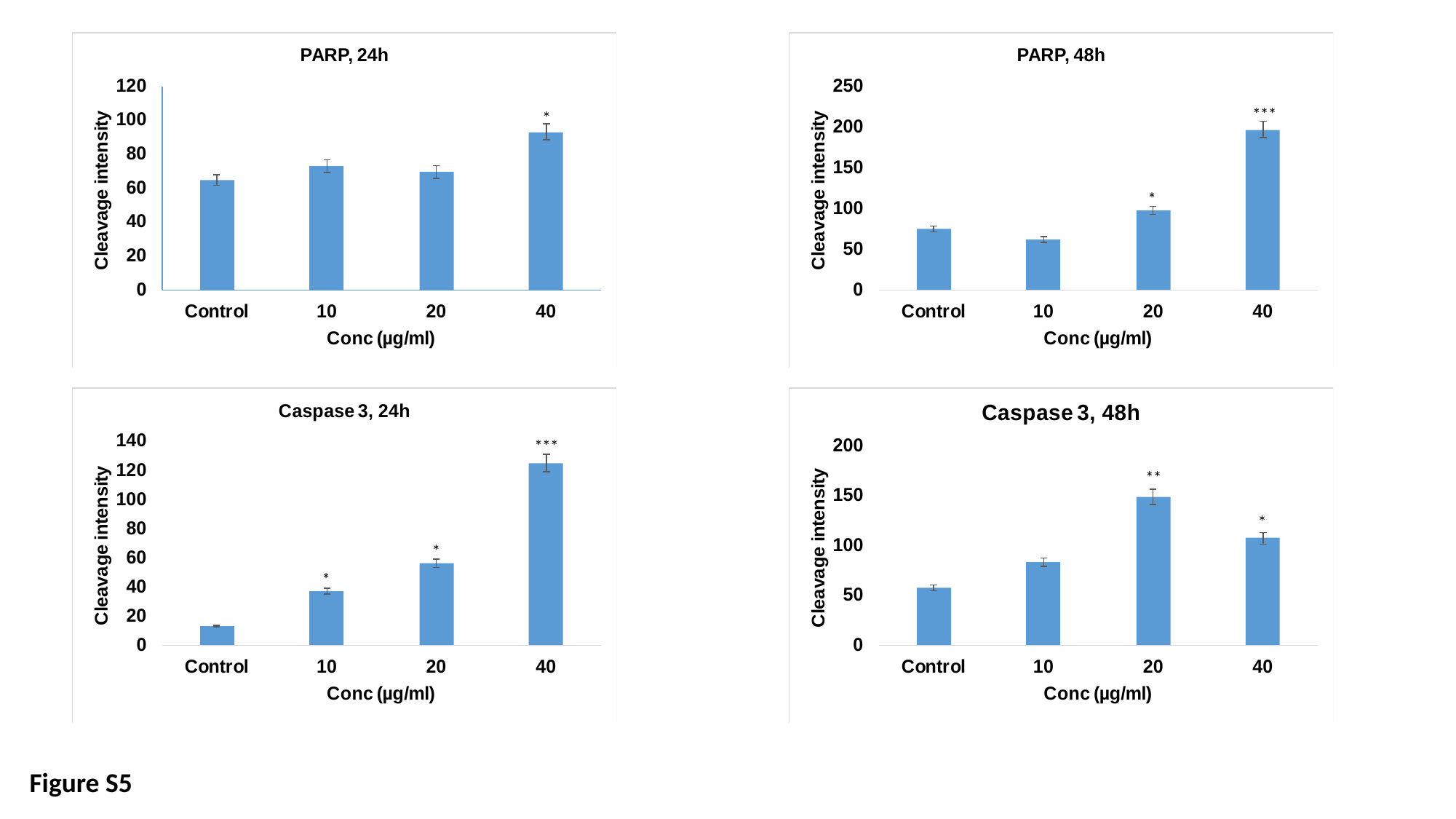

Figure S5

## Slide 7
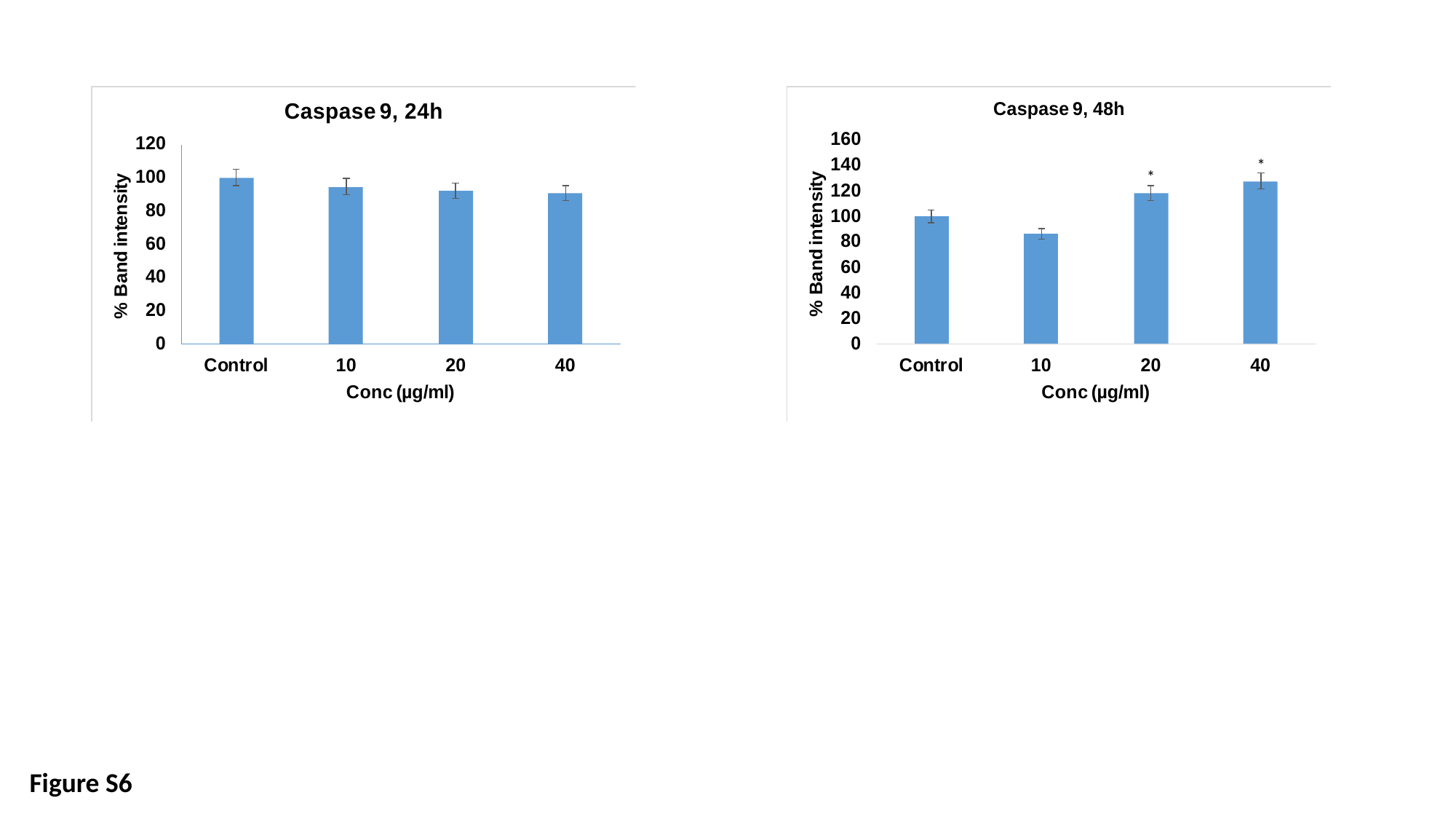

Figure S6

## Slide 8
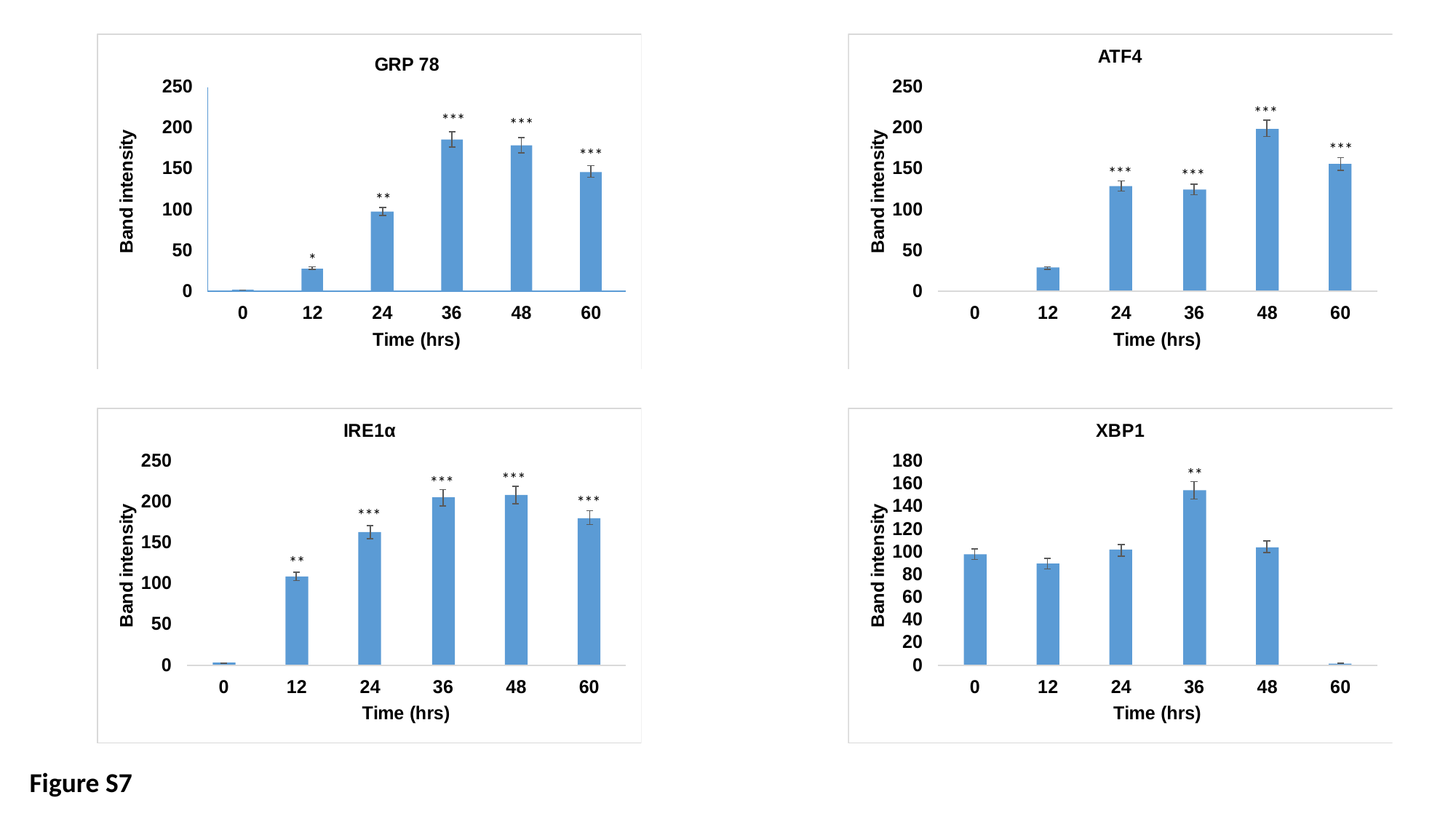

Figure S7

## Slide 9
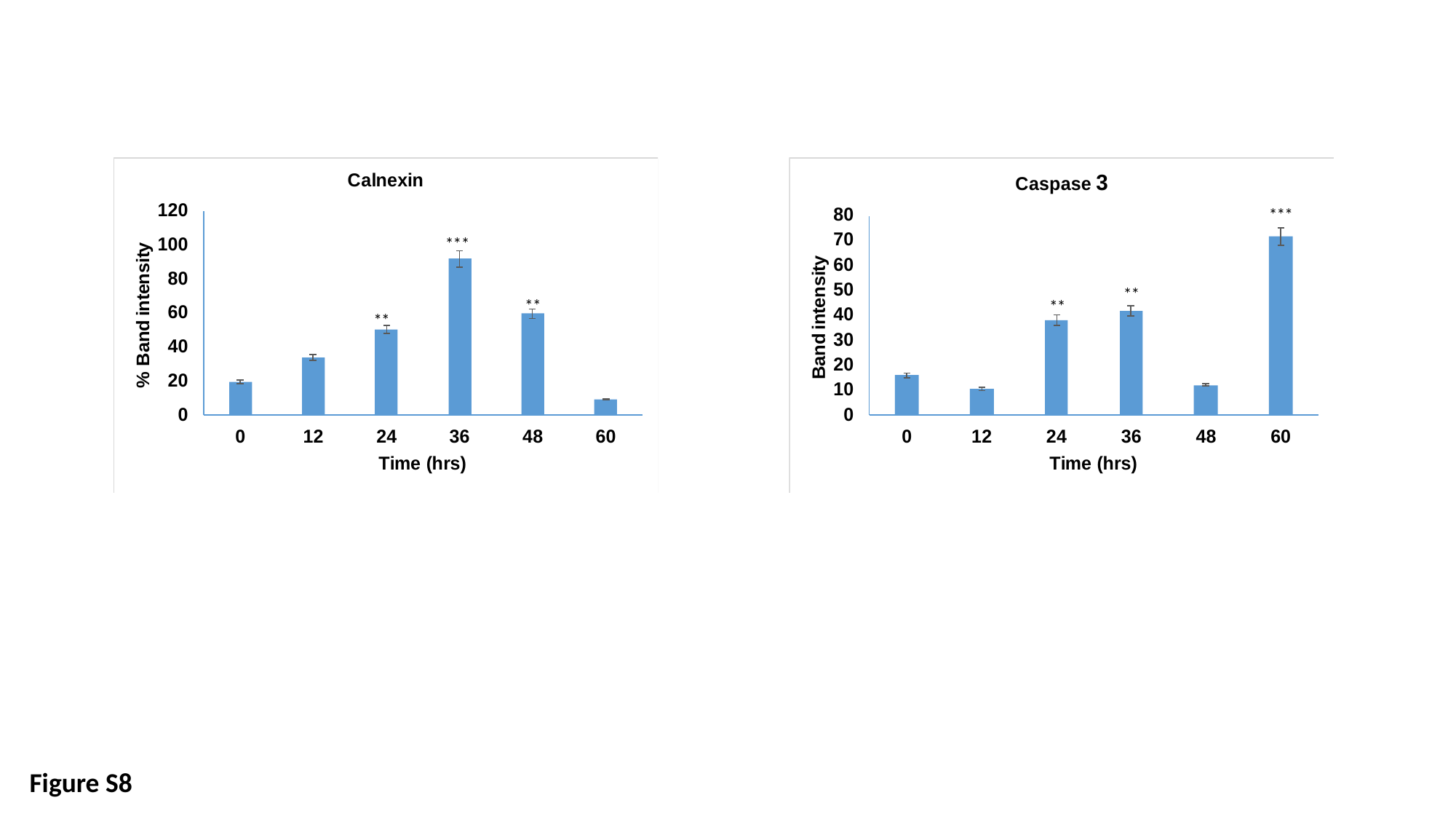

Figure S8

## Slide 10
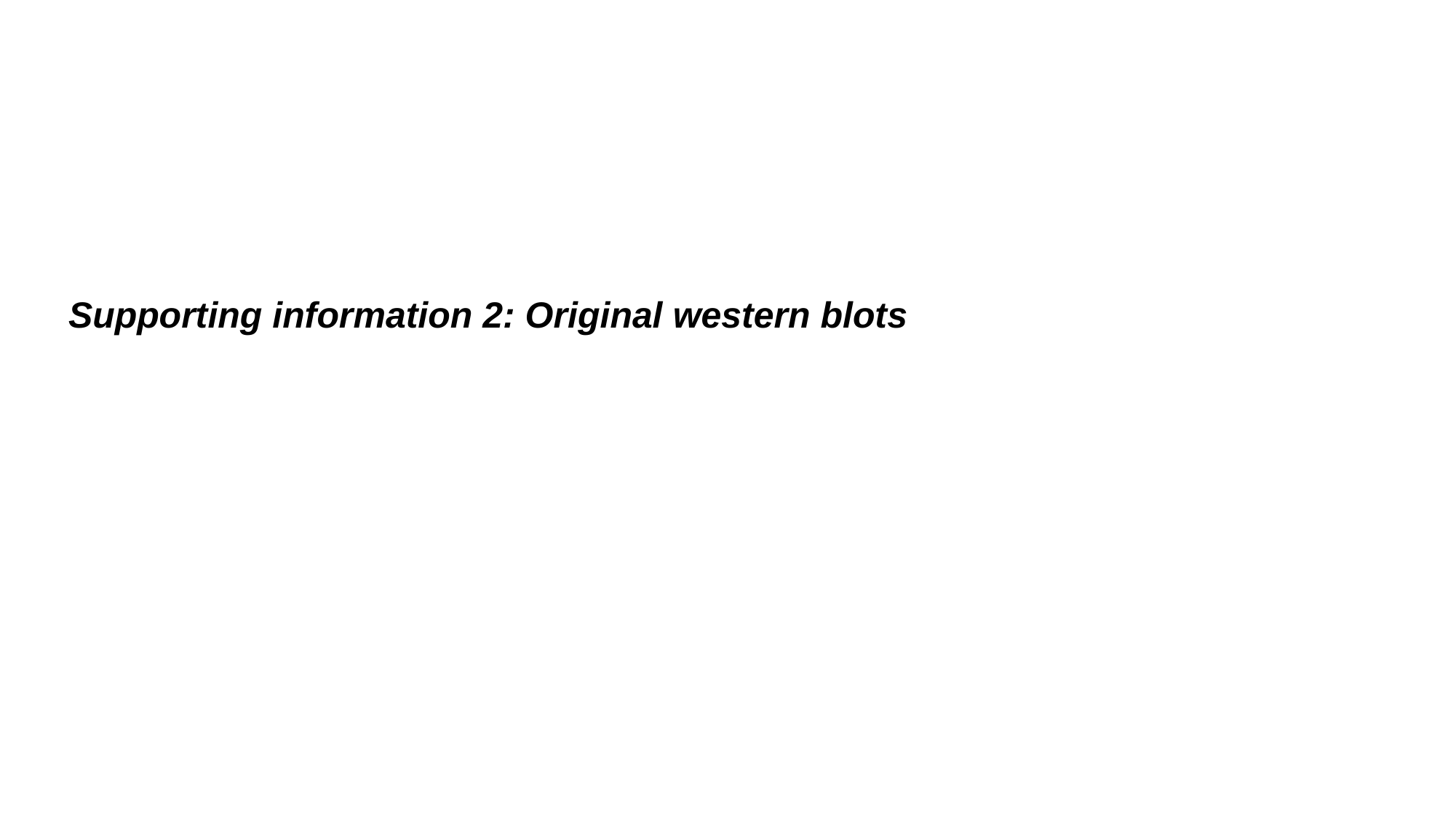

Supporting information 2: Original western blots

## Slide 11
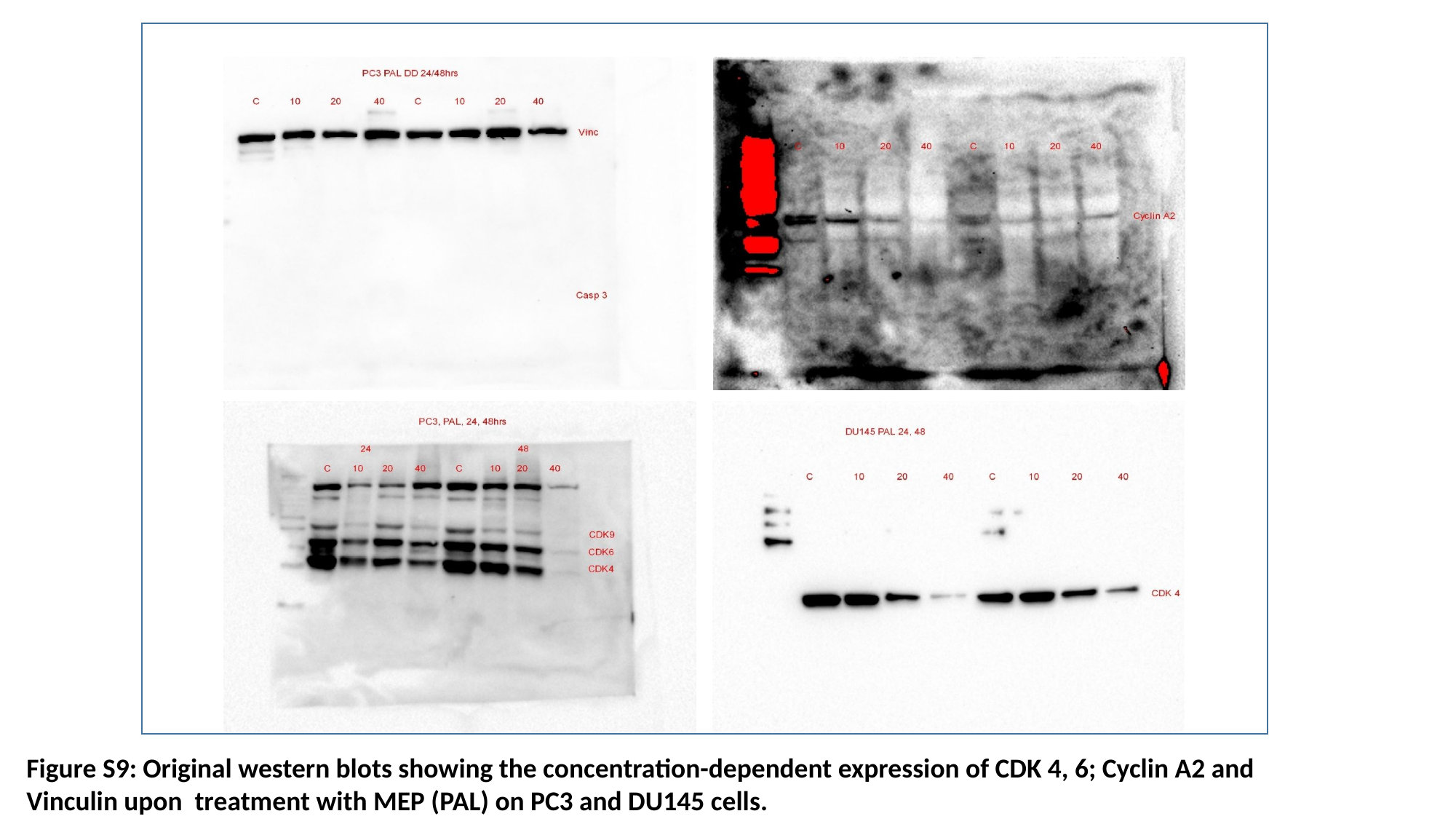

Figure S9: Original western blots showing the concentration-dependent expression of CDK 4, 6; Cyclin A2 and Vinculin upon treatment with MEP (PAL) on PC3 and DU145 cells.

## Slide 12
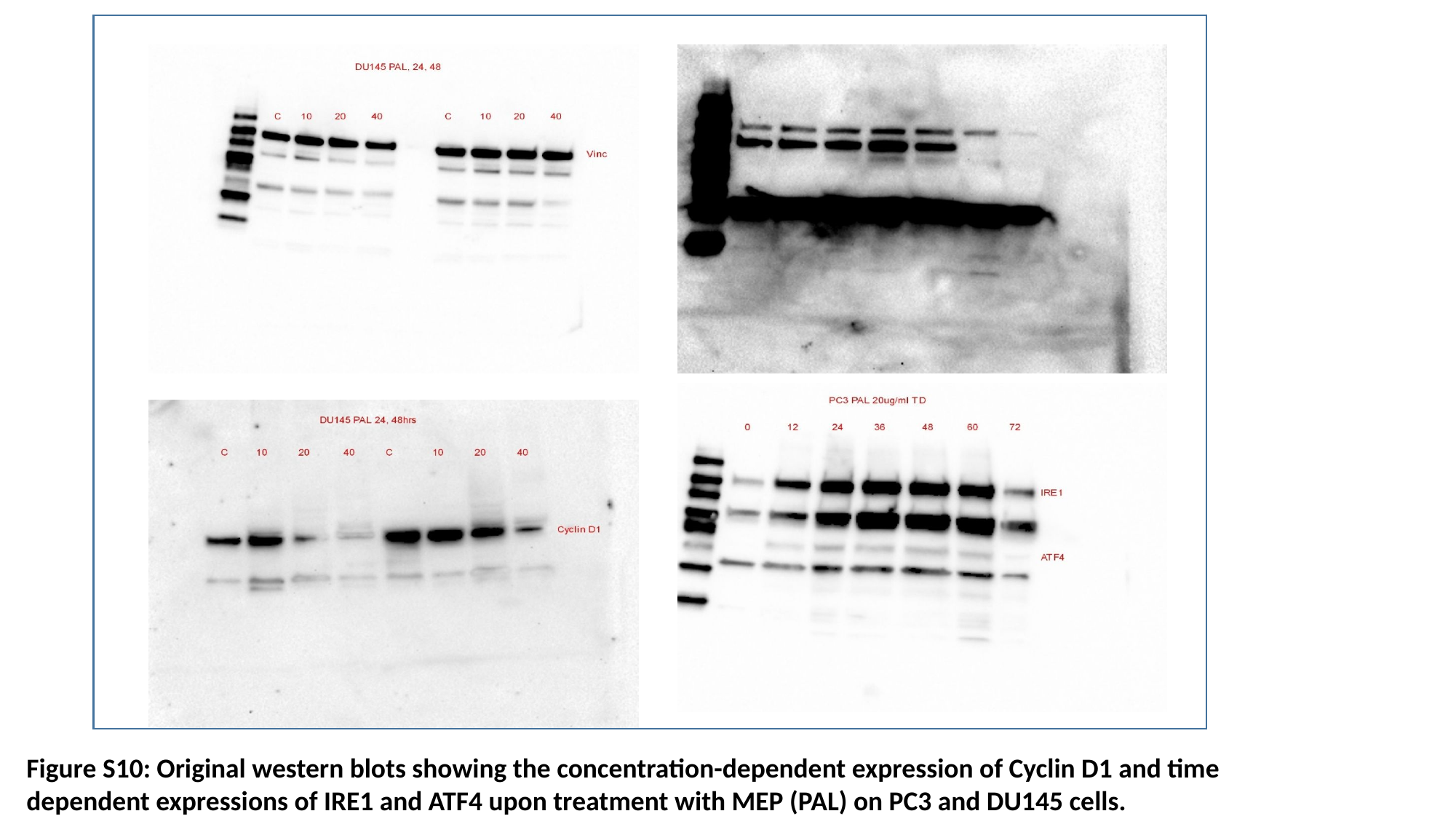

Figure S10: Original western blots showing the concentration-dependent expression of Cyclin D1 and time dependent expressions of IRE1 and ATF4 upon treatment with MEP (PAL) on PC3 and DU145 cells.

## Slide 13
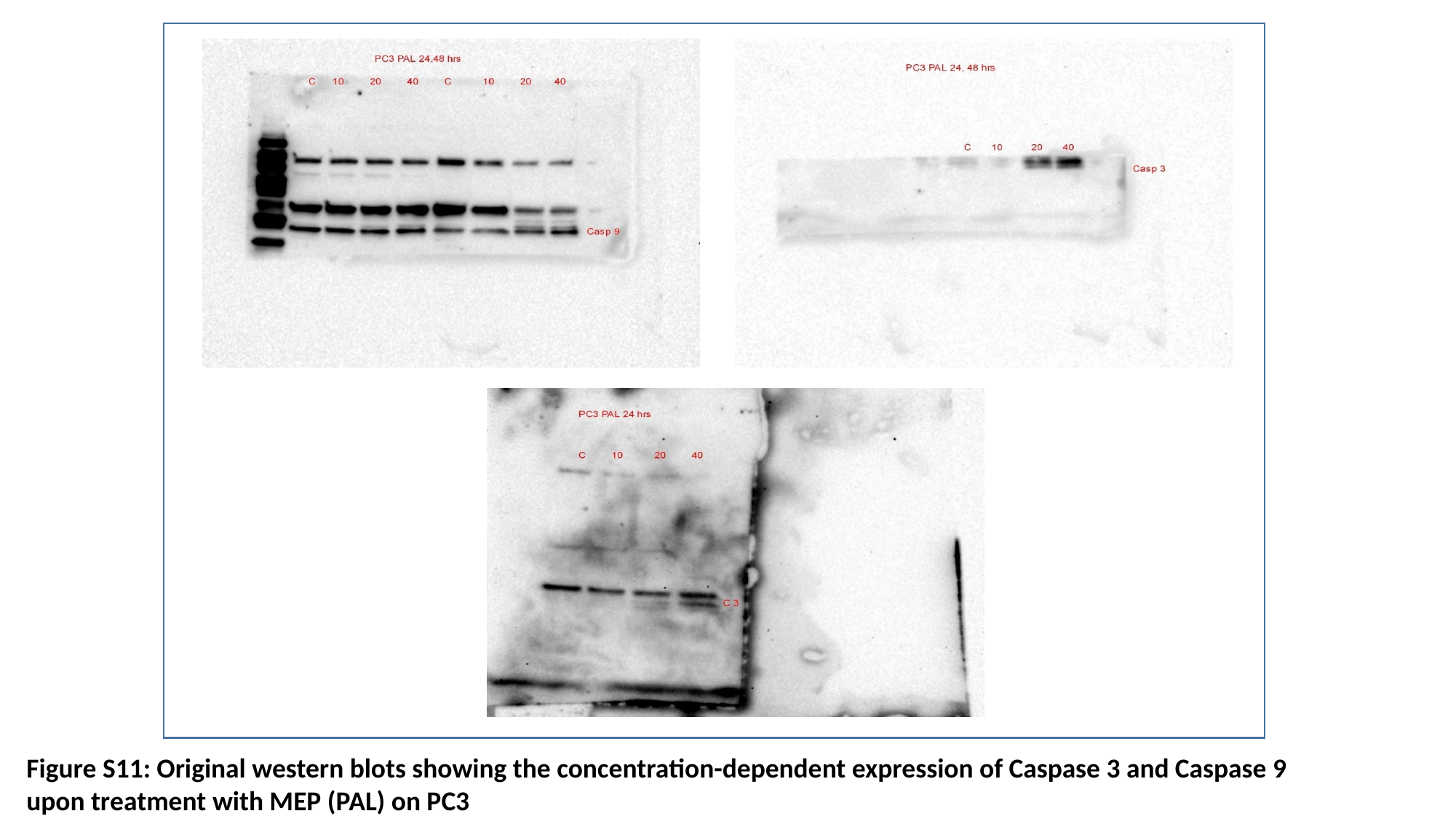

Figure S11: Original western blots showing the concentration-dependent expression of Caspase 3 and Caspase 9 upon treatment with MEP (PAL) on PC3

## Slide 14
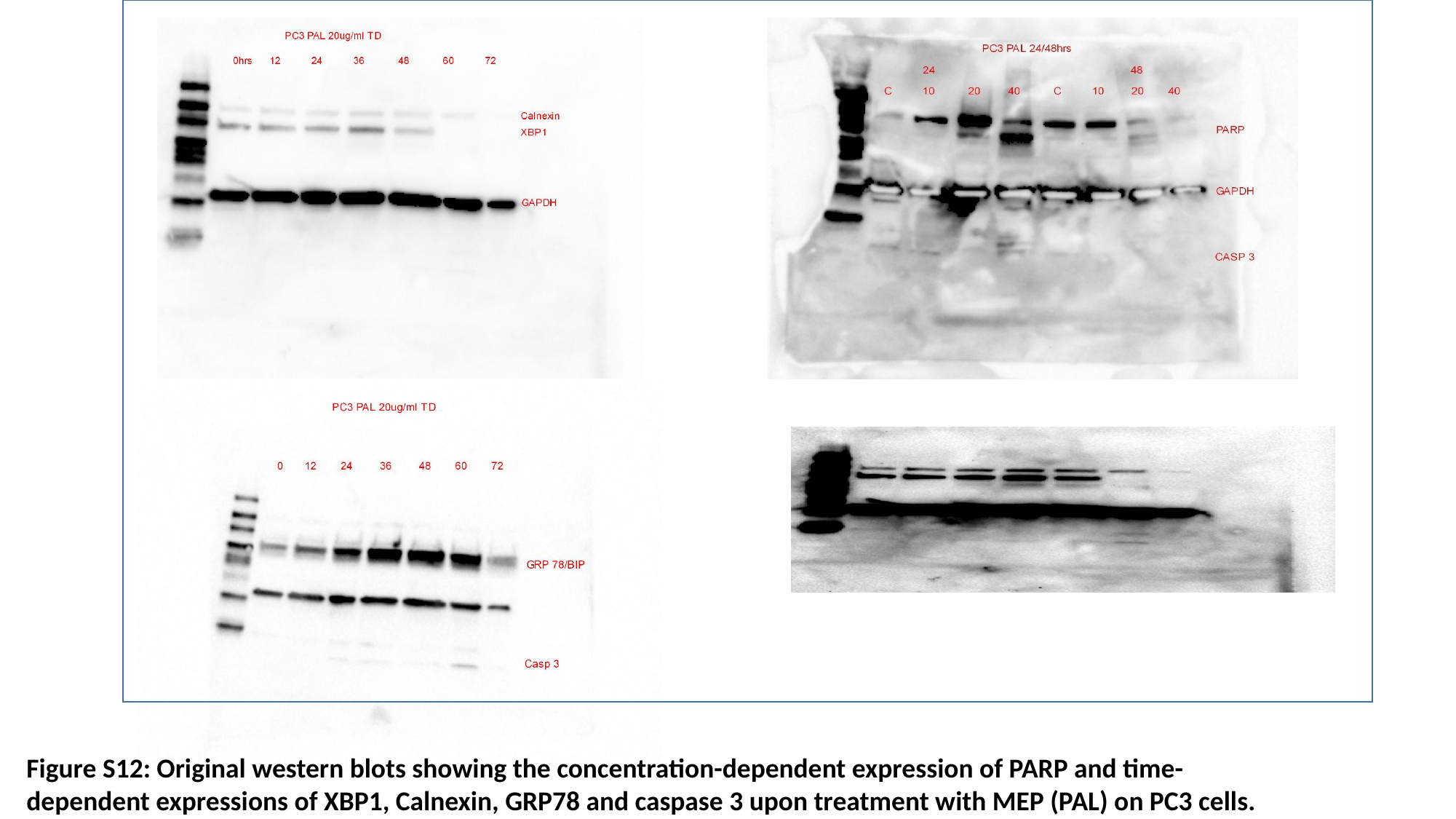

Figure S12: Original western blots showing the concentration-dependent expression of PARP and time-dependent expressions of XBP1, Calnexin, GRP78 and caspase 3 upon treatment with MEP (PAL) on PC3 cells.
